# Developing drought‐smart, ready‐to‐grow future crops

**DOI:** 10.1002/tpg2.20279

**Published:** 2022-11-10

**Authors:** Ali Raza, Muhammad Salman Mubarik, Rahat Sharif, Madiha Habib, Warda Jabeen, Chong Zhang, Hua Chen, Zhong‐Hua Chen, Kadambot H. M. Siddique, Weijian Zhuang, Rajeev K. Varshney

**Affiliations:** ^1^ Key Laboratory of Ministry of Education for Genetics, Breeding and Multiple Utilization of Crops, Oil Crops Research Institute, Center of Legume Crop Genetics and Systems Biology/College of Agriculture Fujian Agriculture and Forestry Univ. Fuzhou 350002 China; ^2^ Dep. of Biotechnology Univ. of Narowal Narowal 51600 Pakistan; ^3^ Dep. of Horticulture, College of Horticulture and Plant Protection Yangzhou Univ. Yangzhou Jiangsu 225009 China; ^4^ National Institute for Genomics and Advanced Biotechnology National Agricultural Research Centre Park Rd. Islamabad 45500 Pakistan; ^5^ Institute of Environmental Sciences and Engineering, School of Civil and Environmental Engineering National Univ. of Sciences and Technology Islamabad 44000 Pakistan; ^6^ School of Science, Hawkesbury Institute for the Environment Western Sydney Univ. Penrith NSW 2751 Australia; ^7^ The UWA Institute of Agriculture The Univ. of Western Australia Crawley Perth 6009 Australia; ^8^ State Agricultural Biotechnology Centre, Centre for Crop and Food Innovation Murdoch Univ. Murdoch WA 6150 Australia

## Abstract

Breeding crop plants with increased yield potential and improved tolerance to stressful environments is critical for global food security. Drought stress (DS) adversely affects agricultural productivity worldwide and is expected to rise in the coming years. Therefore, it is vital to understand the physiological, biochemical, molecular, and ecological mechanisms associated with DS. This review examines recent advances in plant responses to DS to expand our understanding of DS‐associated mechanisms. Suboptimal water sources adversely affect crop growth and yields through physical impairments, physiological disturbances, biochemical modifications, and molecular adjustments. To control the devastating effect of DS in crop plants, it is important to understand its consequences, mechanisms, and the agronomic and genetic basis of DS for sustainable production. In addition to plant responses, we highlight several mitigation options such as omics approaches, transgenics breeding, genome editing, and biochemical to mechanical methods (foliar treatments, seed priming, and conventional agronomic practices). Further, we have also presented the scope of conventional and speed breeding platforms in helping to develop the drought‐smart future crops. In short, we recommend incorporating several approaches, such as multi‐omics, genome editing, speed breeding, and traditional mechanical strategies, to develop drought‐smart cultivars to achieve the ‘zero hunger’ goal.

## Core Ideas

- Drought stress (DS) significantly affects plant growth and development.
- Plants respond and adapt to DS by modifying several physiological, biochemical, and molecular functions.
- Advances in different conventional, biochemical, biotechnological, and breeding techniques reveal plant drought tolerance mechanisms.
- Data from different approaches can be used with speed breeding for drought‐smart, ready‐to‐grow future crops.

**Abbreviations**
ABA: abscisic acid
CAT: catalase
Chl: chlorophyll
CRISPR: Clustered Regularly Interspaced Short Palindromic Repeats
DEG: differentially expressed gene
DS: drought stress
DT: drought tolerance
GAB: genomics‐assisted breeding
GWAS: genome‐wide association studies
JA: jasmonic acid
MDA: malondialdehyde
NAT: natural antisense transcript
POD: peroxidase
QTL: quantitative trait loci
ROS: reactive oxygen species
RWC: relative water content
SA: salicylic acid
SNP: single‐nucleotide polymorphism
SOD: superoxide dismutase
TF: transcription factor
WUE: water use efficiency

## 1 INTRODUCTION

**FIGURE 1:**
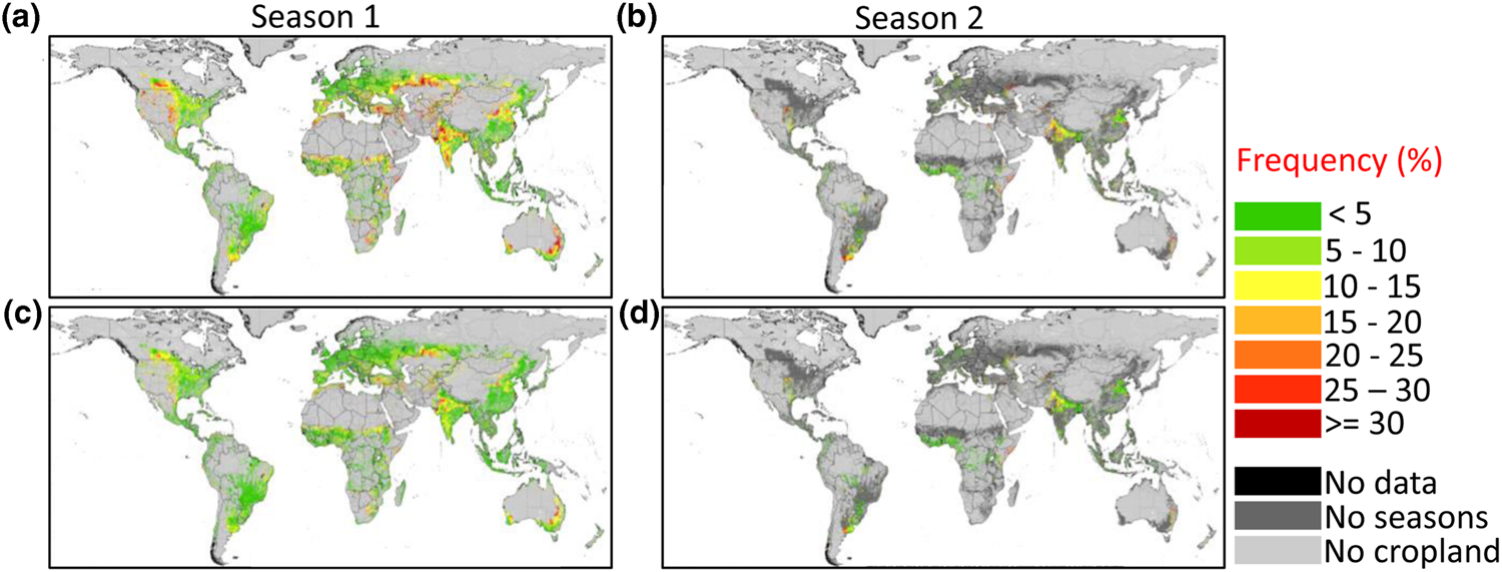
Based on the agricultural stress index, the maps show anomalous vegetation growth and the frequency of historic drought stress in crop zones during the growing Seasons 1 and 2 from 1984–2019. Areas with (a, c) >30% and (b, d) >50% of cropland affected by severe drought stress. *Source*. Global Information and Early Warning System on Food and Agriculture (GIEWS; http://www.fao.org/giews/earthobservation/index.jsp)

Global climate change gives rise to numerous environmental cues including biotic and abiotic stresses, which affect crop productivity (Raza et al., 2019; Farooq et al., 2022). Among them, drought stress (DS) is a destructive natural threat to food security, affecting a substantial fraction of the overall population, mainly those living in arid and semi‐arid areas (Figure 1) (Alamri et al., 2020; Cheng et al., 2021; Rai et al., 2021; Varshney et al., 2021a). Decreased precipitation and altered rainfall models trigger regular DS globally (Figure 1) (Cheng et al., 2021). Consequently, DS limits crop growth and production from germination to maturity (Cui et al., 2020; Tarawneh et al., 2020; Wasaya et al., 2021), thwarting the FAO's goal of ‘zero hunger’. The present stride of crop development is insufficient to feed the growing human population by 2050. Hence, advanced, more stable, and sustainable crop production is necessary to withstand DS (Varshney et al., 2021a). Severe DS hampers crop yield by affecting plant growth, physiology, biochemistry, and reproduction (Yang et al., 2019; Cui et al., 2020; Rai et al., 2021). Plant tolerance and sensitivity to DS relies on various aspects including the drought's impact, duration, and intensity and the plant's genetic potential and development stage (Varshney et al., 2021a). Deeper roots can help plants access soil water under water‐deficient conditions, ultimately increasing yield and production (Soriano & Alvaro, 2019).

Drought stress can usually be described as a prolonged time of irregular, lower‐than‐average natural water accessibility because DS mainly occurs from a substantial shortage in humidity supply as precipitation. Generally, plants are exposed to the DS when (a) the water transfer to the roots is inadequate or (b) the water loss via transpiration is extremely high (Ansari et al., 2019; Cheng et al., 2021; Varshney et al., 2021a). The damage resulting from DS severity is usually unstable, as it is determined by several aspects such as the rainfall forms, moisture availability in soil, and water deficiencies because of transpiration. Consequently, DS hinders crop growth, water–nutrient relations, and photosynthesis and eventually triggers a substantial decline in crop yields (Ansari et al., 2019; Mubarik et al., 2021). Plant responses to DS usually differ from species to species, varying on growth phase and further environmental influences (Araus et al., 2002; Ansari et al., 2019; Cheng et al., 2021).

Plants have evolved many cellular and molecular mechanisms to alleviate DS. A well‐known DS response is abscisic acid (ABA)‐dependent, involving stomatal closure to reduce leaf water evaporation (Li, Yang, Raza et al., 2021). However, being a multiplex trait requiring the stimulation of differentially expressed signaling pathways and molecular responses, the ABA‐mediated response alone cannot mitigate the impact of DS. In plants, the impact of DS increases with induced reactive oxygen species (ROS) formation, decreased antioxidant activity, hormonal imbalance, and expression of stress‐responsive genes. Moreover, knocking out the genes that negatively regulate the DS tolerance mechanisms induced DS tolerance by increasing antioxidant enzyme activity and decreasing hydrogen peroxide (H_2_O_2_) levels (Li, Yang, Raza et al., 2021; Rolly et al., 2020). Plant responses to DS are explained in subsequent sections.

In the field, plants can face single or multiple abiotic stresses at a time. Thus, improving drought tolerance (DT) is of interest to plant breeders. Over the past few years, significant progress has been made in stress mitigation approaches. Hence, we reviewed recent advances in omics tools (genomics, transcriptomics, proteomics, metabolomics, and epigenomics), genome editing, conventional and speed breeding, phytohormone treatments, and agronomic platforms, which help us understand the DS adaptation and tolerance mechanisms in different crop plants and ultimately feed the rapidly growing population and achieve the ‘zero hunger’ goal.

**Core Ideas**

- Drought stress (DS) significantly affects plant growth and development.
- Plants respond and adapt to DS by modifying several physiological, biochemical, and molecular functions.
- Advances in different conventional, biochemical, biotechnological, and breeding techniques reveal plant drought tolerance mechanisms.
- Data obtained from different approaches can be used with speed breeding for drought‐smart, ready‐to‐grow future crops.

## 2 CROP RESPONSES TO DROUGHT STRESS

**FIGURE 2:**
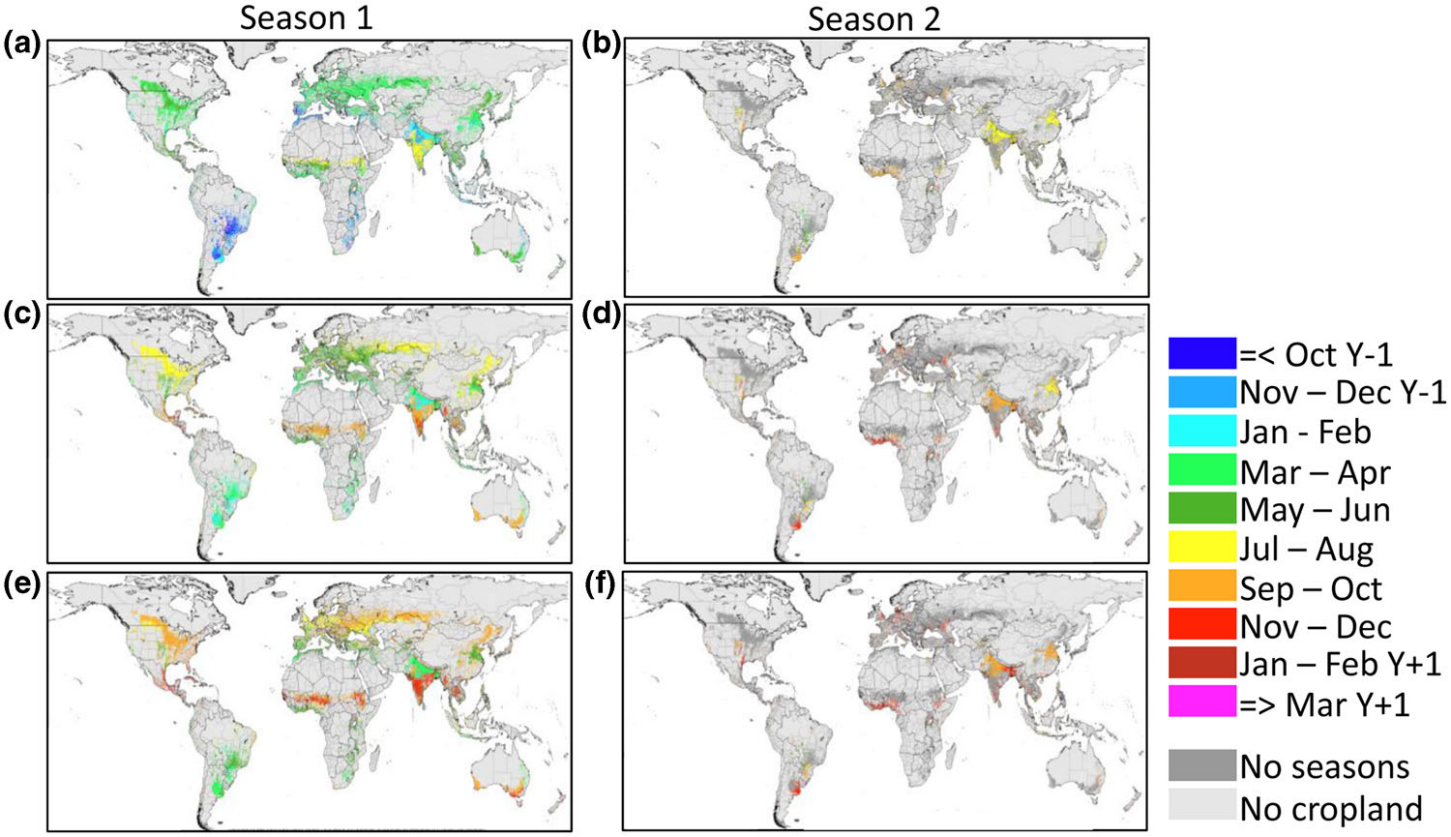
Based on the long‐term average of normalized difference vegetation index (NDVI), the maps show the advancement of vegetation phenology during the growing Seasons 1 and 2. This simplification implies that the crop and pasture phenology is static, and therefore, the growing seasons' progress at a constant rate each year. (a, d) Start of season shows the initial phase of crop development (NDVI reaches 25% of its extreme level), (b, e) peak of the season shows when crop vegetation is completely established (NDVI at its extreme level), and (c, f) end of season shows crops at physiological maturity (NDVI at 75% of its extreme level). This maturity level does not necessarily correspond to the harvest time (yet to get ready for harvest). *Source*. Global Information and Early Warning System on Food and Agriculture (GIEWS; http://www.fao.org/giews/earthobservation/index.jsp)

Plants develop DT through improved morphological, physiological, biochemical, and cellular mechanisms. These improvements induce or suppress gene functions to accumulate osmolytes, upgrade antioxidant defense systems, reduce transpiration, and inhibit the growth of various plant organs such as roots, shoots, and leaves (Alamri et al., 2020; Li, Yang, Raza et al., 2021; Wasaya et al., 2021). Figure 2 shows the growth stage and seasonal impact of DS on crop advancement under DS.

### 2.1 Morphological and physiological responses

#### 2.1.1 Impact of drought stress on crop growth and yield

Drought stress disturbs the functioning of several physiological processes reducing germination, seedling development, and growth. Additionally, DS reduces elongation rate, plant biomass, leaf size, plant height, and relative water content (Handayani & Watanabe, 2020; Malinowska et al., 2020). For instance, DS drastically reduced average leaf area, plant height, shoot length, and leaf dry weight in seven soybean [*Glycine max* (L.) Merr.] cultivars (Rao & Chaitanya, 2019). In another study, DS reduced 1000‐seed weight, biomass, seed weight, and seed number in barley (*Hordeum vulgare* L.) (Tarawneh et al., 2020). Several studies have described the damaging influences of DS on germination and seedling growth as explained in Table 1.

**TABLE 1:** Growth and yield reductions in various crop plants under drought stress

Plant specie	Stress condition	Experimental condition	Effect	References
**Growth**				
Mediterranean barley (*Hordeum marinum* ssp. *gussoneanum*)	40% water‐holding capacity; 6 mo	Field	Reduced average plant height by 20%	Hellal et al. (2019)
Chickpea (*Cicer arietinum* L.)	70% field capacity; 30 d	Field	Reduced average germination rate by 39.5%	Mahmood et al. (2019)
Wheat (*Triticum aestivum* L.)	40–50% field capacity; 6–7 mo	Pot	Reduced heading and filling stage growth by 19.1 and 7.8%, respectively	Li, Lou, Li et al. (2020)
Wheat	25% field capacity; 21 d	Pot	Reduced shoot and grain mass by 30–40%	Mickky et al. (2020)
Rice (*Oryza sativa* L.)	30% PEG6000; 102 h	Field	Reduced heading rate by 85–97%	Wang, Li, Ma et al. (2020)
Tomato (*Solanum lycopersicum* L.)	33% field capacity; 4 mo	Field	Reduced average fruit weight by 78%	Cui et al. (2020)
Wheat	60% field capacity; 2 wk	Greenhouse	Reduced shoot length by 9.65% and root length by 4.29%	Parveen et al. (2021)
Rice	30% field capacity; 4–5 mo	Pot experiment	Reduced plant height by 26%	Tefera et al. (2021)
Maize (*Zea mays* L.)	50% field capacity; 10–20 d	Pot experiment	Reduced plant height by 37.4%	Shemi et al. (2021)
**Yield**				
Maize	50% field capacity; 15 d	Greenhouse	Reduced 100‐kernel weight and plant yield by 85%	Hussain et al. (2019)
Rice	Withholding water; 60 d	Field	Reduced plant yield by 28%	Yang et al. (2019)
Rice	Withholding water; 14 d	Field	Reduced plant yield by ∼50%	Melandri et al. (2020)
Potato (*Solanum tuberosum* L.)	PEG8000; 21 d	Growth room	Reduced average plant yield by 90–95%	Handayani & Watanabe (2020)
Wheat	Amino acid at 3 ml L^−1^; 7 d	Field trial	Reduced grain yield by 3.4%	Haider et al. (2021)
Maize	50% field capacity; 10–20 d	Pot experiment	Reduced grain yield by 30%	Shemi et al. (2021)
Maize	Withholding water throughout the growing season	Rainout shelters	Reduced kernel yield by 15%	Hunter et al. (2021)

Likewise, numerous physiological features regulate the quantity and quality of crop yield. Several physiological processes are affected by DS, reducing yields in most crop plants. The adverse impact of DS on the crop yield is determined primarily by the stress condition (level and duration) and the plant growth phase and condition. Drought stress causes crop yield losses by altering seedling height, reducing shoot length, decreasing hypocotyl fresh weight, limiting water availability to roots, and disturbing the phenological stage (Handayani & Watanabe, 2020; Malinowska et al., 2020). Prolonged exposure to DS affects the flowering and grain filling stages by decreasing grain filling and flower production, leading to drastic losses in crop yield (Farooq et al., 2017; Yang et al., 2019). Some recent examples related to yield losses are listed in Table 1.

#### 2.1.2 Impact of drought stress on crop photosynthesis

Plant photosynthesis is affected by DS, disrupting chemical and enzymatic reactions. Plant organelles responsible for various photochemical reactions change under DS, with long‐lasting effects on photosynthesis (Ansari et al., 2019) including reduced leaf growth, inappropriate functioning of the photosynthetic apparatus, and leaf senescence (Alamri et al., 2020). Stomatal closure under DS decreases the molar fraction of CO_2_ accessible in the chloroplast, making the plant more vulnerable to photo impairment (Zahoor et al., 2017). Under DS, limited soil moisture adversely affects photosynthetic pigments, harms the photosynthetic apparatus, and decreases the concentration of key enzymes responsible for photosynthetic reactions, triggering substantial losses in crop growth and yield (Zahoor et al., 2017; Ansari et al., 2019; Li, Yang, Raza et al., 2021). For example, Marček et al. (2019) studied the effect of water stress on six wheat (*Triticum aestivum* L.) cultivars. The wheat cultivar Ellvis had shown the most pronounced positive changes under DS including decrease in stomatal closure, CO_2_ assimilation, relative water contents, transpiration rate, along with an increase of malondialdehyde (MDA) content. Generally, the negative impacts of DS on photosynthesis are due to constraints in metabolic or CO_2_ diffusion factors (Zahoor et al., 2017).

Moreover, water shortage retards the photosynthetic rate by altering thylakoid membrane function, directly affecting plant growth and production (Huseynova et al., 2007; Aldesuquy et al., 2018). In a recent study, maize (*Zea mays* L.) plants under DS decreased their photosynthetic rate because of increased ROS production and MDA content (Hussain et al., 2019). Drought stress also degrades chlorophyll (Chl) molecules and inhibits Chl biosynthesis, leading to early leaf senescence (Alamri et al., 2020) possibly because of excess ROS generation that induces lipid peroxidation and protein breakdown and alters cellular construction and gene expression in plants. Notably, early leaf senescence reduces photosynthetic capacity and the accumulation of photosynthetic enzymes in reproductive organs, reducing crop yield and quality (Hong et al., 2018; Wang, Lei, Xu et al., 2019). Mustard (*Brassica juncea* L.) plants under DS had improved activity of Chl‐metabolizing enzymes (δ‐aminolevulinic acid dehydratase and porphobilinogen deaminase) and reduced activity of Chl degradation and Chl‐degrading enzymes (chlorophyllase, Chl‐degrading peroxidase, pheophytinase) (Alamri et al., 2020).

#### 2.1.3 Impact of drought stress on crop water and nutrient relations

Relative water content (RWC) decline is the primary consequence of DS on plants; the increase rate of RWC is regulated by numerous aspects such as leaf water potential, transpiration proportion, and stomatal closure. Augmented leaf temperature distracts metabolic roles such as respiration, photosynthesis, ion and nutrient uptake, and amino acid and protein synthesis (Ruehr et al., 2019; Li, Lou, Li et al., 2020; Yu et al., 2021). In addition, DS is frequently associated with oxidative and osmotic stress, causing ion inequality and leading to severe cell membrane structure variations and numerous other cellular functions in plants (Bernardo et al., 2019). Decreased RWC and compromised cell membrane integrity occur in plants grown under DS (Hammad & Ali, 2014). Moreover, DS decreases lipid membranes, damaging cell membranes, which become extra permeable, increasing electrolyte leakage (Petrov et al., 2018). Rodriguez‐Dominguez and Brodribb (2020) reported that moderate DS decreased root and soil hydraulic conductivity, disconnecting the rhizosphere and roots of olive plants. Under DS, proper enzyme functioning and cell turgor pressure are drastically affected, disrupting the transport system and decreasing plant growth and yield (Hatfield & Dold, 2019). Continuous DS (moderate and severe) decreased water use efficiency (WUE) in wheat (Li, Lou, Li et al., 2020).

Similar to other physiological processes, DS significantly affects crop nutrients, especially nitrogen (N), phosphorus (P), potassium (K), silicon (Si), magnesium (Mg), and calcium (Ca), that require water for root uptake (Hussain et al., 2019; Salim & Raza, 2020). Drought stress restricts nutrient translocation in soil by mass flow and diffusion, decreasing plant growth. Nitrogen and water limitations affect crop productivity more than other environmental stresses, with root structure playing a vital role in their uptake into plant vascular systems (Plett et al., 2020). For instance, water‐stressed wheat plants had reduced N, P, and K uptake and translocation because of decreased root volume with reduced Ca, P, and K levels in both roots and shoots (Noman et al., 2018). Hussain et al. (2019) studied the effect of DS on two maize hybrids, reporting that N concentrations in roots > leaves > stems.

Drought stress suppresses the N‐fixation mechanism in legumes by changing crop nodules and decreasing starch content (Plett et al., 2020). Drought stress also changes the accumulation of proteins and amino acids in various crop plants (Umair Hassan et al., 2020). According to Bista et al. (2018), decreased protein uptake in roots is correlated with a reduced nutrient status, hindering crop productivity in maize and barley under DS. Mineral translocation and uptake under DS differ between crop plants. Overall, DS augments N uptake, inhibits P uptake, and has moderate impact on K uptake. Nevertheless, nutrient relations are complicated because of the interactive properties of several nutrients individually and plant physiology as a whole, demanding comprehensive investigations.

### 2.2 Biochemical responses

In general, DS changes the biochemical processes in plants. Sugar is an important energy molecule responsible for the proper functioning of numerous developmental processes. Chinese peony (*Paeonia lactiflora* Pall.) under DS displayed increased soluble sugar and protein contents relative to control plants (Li, Wang, Zhao et al., 2020). Intense sugar activity under stressful environments could be crucial for balancing plant energy levels for normal function. The growth and yield of carrot (*Daucus carota* L.) plants substantially decreased under prolonged DS. Drought stress increased the carotenoid contents of three carrot cultivars (Zhang, Wang, Li et al., 2020). Water stress reduced root fresh weight while enhancing the accumulation of carotenoids and antioxidant activity in radish (*Raphanus sativus* L.) roots (Shafiq et al., 2015). Carotenoids, which are key antioxidants, protect plants from oxidative damage caused by stress. Therefore, it is surmised that carotenoids could act as an antioxidant under DS to ensure normal plant function.

In *Arabidopsis thaliana* (L.) Heynh., MDA content increased under DS at all measured time points (Liu, Li, Li et al., 2020). Moreover, superoxide dismutase (SOD), peroxidase (POD), and catalase (CAT) contents peaked after 17 d of DS. Rapeseed exposed to severe DS (30–40% field capacity) increased MDA and H_2_O_2_ contents by 21% and 71%, respectively, compared with nonstressed plants, and SOD, CAT, and POD activities also increased (Khan et al., 2020). This suggests that plants generate antioxidants for ROS homeostasis, enabling them to withstand DS for a certain amount of time.

Phytohormones are key players in regulating most essential processes during plant growth and development (Mubarik et al., 2021). Phytohormones such as ABA, auxins, cytokinins, gibberellins, ethylene, salicylates, and jasmonates independently normalize plant responses by regulating certain transcription factors and subsequently suppressing or inducing stress‐specific genes. Alternatively, hormone‐to‐hormone or hormone‐to‐sugar crosstalk can facilitate DS responses. For example, endogenous ABA in *Arabidopsis* plants under DS significantly increased compared with control plants (Liu, Li, Li et al., 2020). Similarly, alfalfa (*Medicago sativa* L.) plants subjected to a polyethylene glycol treatment had enhanced endogenous ethylene levels compared with control plants (Defez et al., 2017). Ethylene is a gaseous molecule generally involved in regulating rooting activity against various environmental stresses including DS (Defez et al., 2017). Cytokinin homeostasis is vital for regulating plant responses to DS. Increased cytokinin levels in the roots of young rice (*Oryza sativa* L.) seedling under DS also trigger the increased ROS production, further increasing plant sensitivity to stress (Li, Liu, Li et al., 2020). The level of endogenous melatonin fluctuates under DS (Sharif et al., 2018). For instance, field‐grown maize under DS had higher endogenous melatonin levels than unstressed plants (Huang et al., 2019). Treatment with exogenous melatonin significantly elevated the endogenous melatonin level compared with irrigated plants (Huang et al., 2019). The endogenous gibberellic acid level in maize seedlings under DS decreased initially (3 d postdrought) but recovered to normal levels 6 d after treatment (Zhang et al., 2018). The protective and interactive nature of phytohormones could be a potential strategy for examining the adverse effects of DS on plants.

### 2.3 Molecular responses

Drought stress regulates an array of molecular processes by modulating gene expression (Li, Yang, Raza et al., 2021). Numerous DS‐responsive genes have been identified in various plants. For example, maize plants under DS increased the expression of various transcription factor families, including NAC, bZIP, bHLH, and ERF (Mao et al., 2015; Zhang et al., 2018). Deep sequencing analysis of maize under DS identified *ZmNAC111*, a stress‐specific gene for abundant messenger RNA accumulation, in different tissues (Mao et al., 2015; Zhang et al., 2018). In addition, the transcriptional activity of several hormone‐related genes involved in salicylic acid (SA), gibberellic acid, and ABA metabolism were induced at 6 d after DS treatment in maize (Zhang et al., 2018). Another maize study used a transcriptomics analysis to better understand the molecular mechanisms of ‘sink’ or ‘source’ organs and their impact on yield under DS, revealing that ABA‐ and NAC‐mediated signaling pathways, protein folding, and osmotic protective substance synthesis were all common DS‐responsive elements among maize ears, kernels, and ear leaves (Wang, Liu, Zhang et al., 2019). The *SlGRAS4* gene upregulated transcriptional activity in response to tomato (*Solanum lycopersicum* L.) under DS (Liu, Wen, Shi et al., 2020). Further molecular analysis revealed that *SlGRAS4* binds directly to the class III *SnRK2s* gene (Liu, Wen, Shi et al., 2020), a crucial regulator of ABA signaling (Duarte et al., 2019). At the protein level, the *SlSnRK2.4* gene further interacts with downstream genes (*SlAREB1* and *SlAREB2)* of the ABA signaling pathway (Liu, Wen, Shi et al., 2020), suggesting that *SlGRAS4* can be a potent regulator of mediating plant response to DS.

Several transcription factors, including the HD‐ZIP gene family (Sharif et al., 2021), are major plant response regulators to DS, maintaining physiological and biochemical processes (Sharif et al., 2021). For instance, *AtHB7* and *AtHB12*, two paralogous genes, are induced under ABA and water stress to regulate stomata closure (Perotti et al., 2017; Gong et al., 2019). *SiHDZ13* and *SiHDZ42* genes showed upregulated transcriptional activity in sesame (*Sesamum indicum* L.) under prolonged DS (Wei et al., 2019). Wheat *Tahdz4‐A* expression significantly increased under DS, conferring its responsive nature to DS (Yue et al., 2018). *HD‐ZIP* genes are also involved in modulating the ABA‐independent DS response in many plant species (Sharif et al., 2021). The ABA‐independent pathway genes, such as *DREB* genes belonging to AP2/ERF (Sharif et al., 2021), are key plant response regulators to DS. In addition, *TaHDZipI‐3* and *TaHDZipI‐4* induced significantly under DS could be vital for improving plant responses to DS in wheat (Yang et al., 2020). The expression of *HDZI‐3* and *HDZI‐4* promoters were analyzed after drought treatment, and both had elevated transcriptional activities (Yang et al., 2020). Therefore, *HDZI‐3* and *HDZI‐4* were used to develop *DREB/CBF* transgenic plants (Yang et al., 2020); under the constitutive *HDZI‐3* and *HDZI‐4* promoters, these plants significantly increased messenger RNA accumulation of the *DREB/CBF* gene and grain yield in wheat and barley (Yang et al., 2020).

## 3 MANAGEMENT STRATEGIES

### 3.1 Omics tools

**FIGURE 3:**
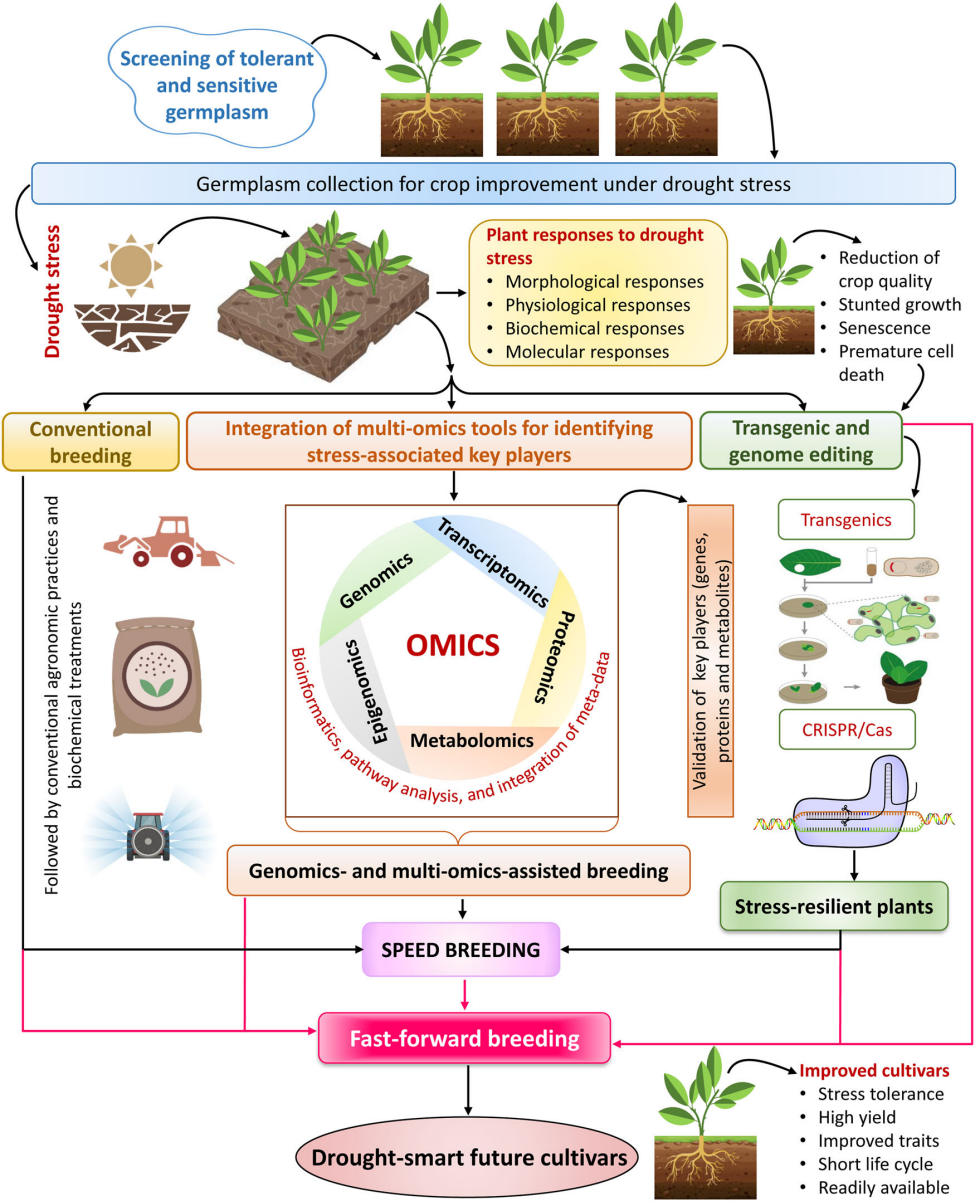
Proposed scheme for developing drought‐smart future crop plants. Fast, accurate, and targeted exploitation of various plant traits is essential. Different omics approaches, including genomics, transcriptomics, proteomics, metabolomics, and epigenomics, can deliver a set of stress‐associated key players (gene, proteins, and metabolites), which can be validated using transgenics or genome editing (mainly CRISPR/Cas system) approaches. The obtained stress‐smart plants could be introduced to modern breeding methods to improve cultivars. Among them, speed breeding has revolutionized plant breeding to the next level, with five to six generations in a year (Watson et al., 2018). Integrating speed breeding with new‐age genomic breeding methods could ease the long‐standing bottleneck of prolonged crop breeding cycles (Watson et al., 2018). Fast‐forward breeding schemes could be coupled with other breeding schemes to enhance sustainable agricultural production under stress (Varshney et al., 2021b). Notably, fast‐forward breeding can maintain genetic diversity in breeding programs to increase genetic gains from breeding revolutions

After the successful sequencing of numerous plant genomes, significant efforts have been made to use this information to boost plant productivity under water scarcity. In response to environmental stressors, plant genome, transcriptome, proteome, metabolome, and epigenome studies have revealed valuable information on the mechanisms driving cellular processes in response to different stresses and insights into complex physiology–cell–environment interactions (Varshney et al., 2018, 2020; Pazhamala et al., 2021). Furthermore, current state‐of‐the‐art omics approaches have identified differentially expressed genes (DEGs) for use as biomarkers to develop drought‐resilient crop plants (Raza et al., 2021; Varshney et al., 2021d). Therefore, the emerging omics field has significantly enhanced our understanding of plant physiology and gene function in response to DS (Figure 3; Table 4).

#### 3.1.1 Genomics‐assisted breeding for drought tolerance in plants

Deployment of genomics‐assisted breeding (GAB) approaches, such as marker‐assisted selection, haplotype‐based breeding uses tightly linked molecular markers associated with the desirable trait to select plants at an early stage for breeding (Varshney et al., 2021d). Genome mapping, as with biparental or quantitative trait loci (QTL) mapping and genome‐wide association mapping, requires a high throughput genotyping platform that screens the whole genome with higher mapping resolution using molecular markers, which can be used in breeding programs (Figure 3) (Salgotra & Stewart, 2020; Scott et al., 2020; Varshney et al., 2021d).

##### QTL mapping

Linkage or QTL mapping offers genetic insight into physiological and agronomic traits in the biparental population under DS (Honsdorf et al., 2017). Thus, it is used to recognize genomic regions with variable contributions to the stress tolerance phenotype. For instance, the wheat RAC875/‘Kukri’ population is often used for genetic analysis for DS tolerance, as the QTL on chromosome 3BL was studied at 21 different environments of Australia and Mexico. The parent RAC875 improves yield by 12.5% in heat and drought stress environments, whereas grain yield increased by parent Kukri up to 9% at favorable environments (Bonneau et al., 2013). Another study identified 11 QTLs in the same population under DS during a 2‐yr trial (Salapour et al., 2020). Three QTLs for grain yield were identified under both normal and DS condition from which the QTL on chromosome 3D explains 6.47% variation, whereas on chromosomes 4B and 6A major epistatic effect was identified that explains 14.84% phenotypic variation with 5.7 logarithm of the odds score (Salapour et al., 2020).

Chickpea (*Cicer arietinum* L.), a rich source of protein, carbohydrates, and minerals and therefore provides a major supply of nutrients in vegetarian diets particularly in developing countries (Varshney et al., 2019). In chickpea, several studies have been carried out for fine mapping the “*QTL‐hotspot*” region to identify the candidate genes linked with DS tolerance and development of closely associated markers (Varshney et al., 2013, 2014; Jaganathan et al., 2015; Kale et al., 2015; Bharadwaj et al., 2021). For instance, by employing phenotyping data for 20 DS tolerance‐linked traits collected in one to seven seasons at one to five locations in India and genotyping data for 241 simple sequence repeat loci on one intraspecific population (ICC 4958 × ICC 1882), the authors recognized a “*QTL‐hotspot*” region harboring 12 QTLs for 12 DS tolerance‐linked traits explaining up to 58.20% percentage variance explained (Varshney et al., 2014). In another study, the introgression of this “*QTL‐hotspot*” region in ‘JG 11’ (an elite cultivar) has improved root traits and DS tolerance (Varshney et al., 2013). In a recent study, these authors reveal that the introgression of the “*QTL‐hotspot*” region into three elite chickpea cultivars from India (‘Pusa 372’, ‘Pusa 362’, and ‘DCP 92‐3’) improves DS tolerance and seed yield under DS conditions (Bharadwaj et al., 2021). They also proposed that superior introgression lines used in various genetic backgrounds can be verified for likely release as developed varieties in India. The summary of some representative studies is presented in Table 2.

**TABLE 2:** Summary of some quantitative trait loci (QTL) mapping‐related experiments under drought stress in different plant species

Plant specie	Cross	Markers	Approach, linkage map	QTL or gene	No. of lines or accessions used	Chromosomal location	Key observations	References
Chickpea (*Cicer arietinum* L.)	ICC 4958 × ICC 1882 and ICC 283 × ICC 8261	241 and 168 SSR loci on eachf intra‐specific population	CIM in Win QTL cartographer	45 main effect and 973 epistatic QTL	232 and 234 RILs of each population	LG04	“*QTL‐hotspot*” region harboring 12 QTL for 12 DT‐linked traits explaining up to 58.20% PVE on CaLG04 containing seven SSR markers	Varshney et al. (2014)
Wheat (*Triticum aestivum* L.)	‘Kukri’ × RAC875	1333 loci of integrated SSR–DArTs–SNP linkage map	ICIM and ICIM‐EPI in ICiMapping v4.0 software	37 putative main effect and 149 epistatic QTL	220 double‐haploid lines	1A, 2A, 3A, 4A, 5A, 3B, 4B, 5B, 7B, 1D, 2D, 3D	DH‐R118, DH‐R172, DH‐R250 lines identified for MAB due to high yield, STS, and drought tolerance; RAC875 alleles had major contribution to DT	Salapour et al. (2020)
soybean [*Glycine max* (L.) Merr.]	‘Zhonghuang 35’ × ‘Jindou 21’	8078 SLAF‐seq	ICIM‐ADD in ICiMapping v4.1 software	23 QTL	234 RIL	2, 4, 6, 7, 10, 17, 19	Plant height and seed weight used as indicators; seven QTL identified for plant height and five QTL linked to seed weight; three QTL (qPH6/qSWPP6, qPH17/qSWPP17, qPH19–3/qSWPP19) linked to DT	Ren et al. (2020)
Rice (*Oryza sativa* L.)	CR 143‐2‐2 × ‘Krishnahams’	77 SSR	ICIM v4.0 and ICIM v4.0	3 QTL	190 RIL	1, 3	Ten traits were studied and identified three QTL as qRCC1.1; qCHLa1.1, and qPRO3.1 for relative chlorophyll content, chlorophyll, and proline content under DT	Barik et al. (2020)
Common bean (*Phaseolus vulgaris* L.)	‘Portillo’ × ‘Red Hawk’	810 polymorphic SNP markers	CIM in Win QTL cartographer	8 QTL	97 RIL	Pv01, Pv02, Pv03, Pv04, Pv06, Pv11	Under DS, six QTL identified for grain yield per plant at chromosome Pv01, 02, 03, 04, and 06; Two candidate genes at Pv03 identified for MAB	Dramadri et al. (2019)
Common bean	KATB1 × GLP2	1,578 SNP and 374 polymorphic	CIM in Win QTL cartographer	23 QTL	102 F_2_ population	Pv01, Pv02, Pv03, Pv04, Pv08	Two QTL identified for grain yield; two QTL linked to seed yield on chromosome Pv01, Pv02; Pv02 chromosome‐linked to QTL for DT	Langat et al. (2020)
Oilseed rape (*Brassica napus* L*.)*	RP04 × ‘Ag‐Outback’	18,851 DArT marker	Baseline model in R software using ASReml v3.0 library	53 QTL	156 double‐haploid + 2 parent + 21 check = 188	25 genomic regions; significant QTL on A06, A10, C04	Nine QTLs linked to seed yield on eight chromosomes with drought tolerance index	Raman et al. (2020)
Horsegram (*Macrotyloma uniflorum* L.)	HPK4 × HPKM249	211 markers (157 SSR, 39 random amplification of polymorphic DNA, 8 intersimple sequence repeats, 7 conserved ortholog set)	CIM in QTL Cartographer v2.5	5 QTL	190 RIL	LG1, LG4, LG6, LG7	qDFW01, qDFW02, qDTM01, qRL01, qNSPP01 identified for DT, explaining 7.2–67.3% phenotypic variance	Chahota et al. (2020)
Groundnut (*Arachis hypogaea* L.)	‘TAG 24’ × ICGV 86031)	58K SNP by Affymetrix ‘Axiom‐Arachis” array’	ICIM‐ADD; ICIM‐EPI in ICIM v4.1.0.0	129 QTL to drought tolerance	309 RIL	A01, A02, A03, A04, A05, A07, A09, B01, B09	19 QTL with major effect on DT with 10–33.0% PVE. Major DT‐related linked traits are hulm, pod, and 100‐seed weights	Pandey et al. (2021)
Rapeseed (*Brassica napus* L.)	‘KenC‐8’ × N53‐2	17,978 SNPs and 101 non‐SNP markers (SSR and STS)	CIM by QTL Cartographer 2.5 software	39 QTL	300 double‐haploid lines with two parents	A and C genome	18 QTL linked to drought susceptibility index of four traits (germination percentage, root length, shoot length, and root/shoot length ratio). Three drought stress tolerance loci found (BnaC03g32780D, BnaC03g37030D, BnaC09g27300D); Noval QTL found on genomic region A06, C01, and C09	Gad et al. (2021)
Jute (*Corchorus capsularis* L.)	‘Aidianyehuangma’ × ‘Huangma 179’	69,446 SLAF‐seq	ICIM mapping in IciMapping 4.0	27 QTL	100 RIL	Chr 1, 2, 3, 4, 5, 6, 7, 8, 9	Three candidate genes identified related to salt tolerance and DT: CCACVL1‐12635, CACVL1‐15402. CCACVL1‐23216	Ibrahim et al. (2021)
Sesame (*Sesamum indicum* L.)	‘Zhushanbai’ × ‘Jinhuangma’	466,911 SNPs; 1,354 bin markers	CIM in QTL cartographer v2.5	34 QTL	180 RIL	10 chromosomes	13 stable QTL identified; eight linked to water stress conditions at Chr 1, 4, 5, 7, 8, and 12	Liang et al. (2021)

*Note*. CIM, composite interval mapping; DArT, diversity arrays technology; DT, drought tolerance; DS, drought stress; ICIM, inclusive composite interval mapping; ISSR, inter simple sequence repeat; MAB, marker‐assisted breeding; PVE, percentage variance explained; RIL, recombinant inbred lines; SLAF‐seq, specific locus amplified fragment sequencing; SNP, single‐nucleotide polymorphism; SSR, simple sequence repeat; STS, sequence‐tagged sites.

##### Genome‐wide association studies

Genome‐wide association studies (GWAS) can identify contributory alleles for specific traits that can be used in GAB to develop DS‐tolerant crop plants (Table 3) (Habib et al., 2020; Varshney et al., 2020). Huge genetic diversity was found among elite wheat cultivars under drought and heat stress; plant grain yield had a linear relationship with the DS index, and 11 significant marker–trait associations on chromosomes 1B, 2A, and 7D were associated with plant yield (Abou‐Elwafa & Shehzad, 2021). Maize production is frequently affected by increased frequency and intensity of DS (Lobell et al., 2014). In maize, GWAS analysis identified 27 significant single‐nucleotide polymorphisms (SNPs) linked to enhanced seminal root length playing a crucial role in DS tolerance (Guo, Li et al., 2020).

**TABLE 3:** Summary of some genome‐wide association studies (GWAS) under drought stress in different plant species

Plant specie	Markers	Approach, software	No. of lines or accessions used	Chromosomal location	No. linked SNPs or QTL	Key observations	References
Wheat (*Triticum aestivum* L.)	90K Illumina Infinitum SNP array; 15,737 SNPs	MLM in Tassel software	290 lines of wheat association mapping initiative	2A, 3A, 5A, 6A, 7A, 1B, 2B, 4B, 5B, 6B,7B, and 7D	205 MTA; 89 at drought stress SNP	24 MTA found for days to heading, 11 for plant height, three for tiller number plant^‐1^, 34 for shoot length, 11 for plant grain yield, and six for 100‐grain weight	Abou‐Elwafa & Shehzad (2021)
Wheat	660K SNP array; 395,681 SNPs	GLM and MLM in Tassel v5.0 and FarmCPU	277 winter wheat lines	1A, 1B, 1D, 2A, 2B, 2D, 3A, 3B, 3D, 4A, 4B, 4D, 5A, 5B, 5D, 6A, 6B, 6D, 7A, and 7D	189 SNPs; 69 under DS	Eight SNPs linked to grain yield per plant under DS	Li, Mao, Wang et al. (2019)
Maize (*Zea mays* L.)	Maize SNP50 BeadChip; 43,252 SNPs	MLM in Tassel v5.0	209 diverse inbred lines	22 genomic regions under water‐stressed conditions (WS)	62 significant SNPs with seminal root length and 27 under WS	Seminal root length GWAS depicts 27 SNPs under WS and 506 unique candidate genes	Guo, Li et al. (2020)
Soybean [*Glycine max* (L.) Merr.]	SoySNP6k iSelect BeadChip; 5,361 SNPs	MLM in Tassel v5.0	259 chinese cultivars	13 chromosomes: 4, 5, 6, 7, 9, 11, 12, 13, 14, 17, 18, 19, and 20	15 SNPs	15 QTL linked to three DT indices. Two QTL on each chromosome 12 and 20. QTL on chromosome 11, 17 and 20 strongly linked to DT	Liu, Li, Gou et al. (2020)
Upland cotton (*Gossypium hirsutum* L.)	2,060,458 SNPs	FaSt‐LMM v2.02; PLINK software v1.90b6.8	200 upland natural population	A02, A04, A12, A13, D01, D09, D10, D11, and D13	622 SNPs; 15 QTL	15 QTL within 167 DEGs with four i‐traits; 16,827 DEGs linked to DS	Liu, Li, Gou et al. (2020)
Barley (*Hordeum vulgare* L.)	9K Illumina iSELECT geneotyping BeadChip; 7,865 SNPs	Mixed model MTA; Genstat18 software and PhenoGram Plot	183 spring barley EcoSeed panel	1H, 2H, 3H, 4H, 5H, 6H, and 7H	97 MTA; 28 for drought	2H, 6H, and 7H SNP confirmed for drought tolerance that colocalized with 10 putative genes, including RNA‐binding protein, plant respiratory burst oxidase homologs family, and carotenoid cleavage dioxygenase1	Tarawneh et al. (2020)
Barley	9K Illumina SNP Chip	MLM and GLM in GAPIT R‐package	121 spring barley accessions	1H, 2H, 3H, 4H, 5H, 6H, and 7H	101 significant SNPs	Two drought‐related genes identified at 2H; Genes expressed in grains, spikes, spikelets, and leaves correlated with DT	Thabet et al. (2020)
Upland cotton	Illumina HiSeq 2500; 473,516 SNPs	MLM in GAPIT R‐Package	550 RIL multiparent advanced generation intercross population	A01, A04, A08, A09, A12, A13, D02, D03, D05, D06, and D08	43 QTL; 20 for DT	13 QTL linked to PH and seven to dry shoot weight under DT; nine QTL linked to both DT and salt tolerance	Abdelraheem et al. (2021)
Maize	Image‐based traits (i‐traits) GWAS; 26,910 I‐traits	Tassel v5.0 uncompressed P3D model	368 lines	All chromosomes	4,322 significant locus trait associations	1,529 QTL and 2,318 candidate genes; 15 i‐traits are potential markers for drought. Two new genes, *ZmcPGM2* and *ZmFAB1A*, linked with i‐traits and DT	Wu et al. (2021)
Wheat	Illumina 90K iSelect Wheat SNP assay; 81,587 SNPs	MLM with PC + kinship in Tassel v5.0	361 genotypes	1A, 3A, 3B, 4B, 4D, 5B, 6A, and 6B	69 QTL on all chromosomes except 5D	16 QTL for drought; six novel QTL at 3D, 4A, 5B, 7A, and 7B; 5B, 6B, and 4B important for DT	Rabbi et al. (2021)

*Note*. DEG, differentially expressed gene; DT, drought tolerance; DS, drought stress; GLM, generalized linear model; MLM, mixed linear model; MTA, marker–trait association; QTL, quantitative trait loci; SNP, single‐nucleotide polymorphism.

Soybean GWAS analysis under DS revealed 15 QTL on 13 chromosomal regions explaining 5.81% of the phenotypic variation. Three QTL on chromosomes 11 (ss247449682), 17 (ss249472124), and 20 (ss250606162) linked to two DS tolerance traits from radical length, radical weight, and germination rate offer a genetic resource for GAB (Liu, Li, Gou et al. (2020). The MAGIC population of cotton, comprising 550 recombinant inbred lines and 11 upland parents, were genotyped using >47,000 SNP markers; 20 and 23 QTL were identified with drought and salt tolerance, respectively. Notably, 21% of the identified QTL were common for drought and salt tolerance, with 53 candidate genes associated with other abiotic stresses in cotton (Abdelraheem et al., 2021). Recently, Ahmed et al. (2021) studied the DS tolerance of 138 diverse wheat seedlings using 407 Diversity Arrays Technology markers and identified 104 significant QTL. Marker WPT‐2356 was linked to the drought susceptibility score for all traits. Moreover, 264 accessions of rapeseed (*Brassica napus* L.) were studied for water loss ratio. The GWAS analysis identified 139 linked SNPs with water loss ratio, of which, 13 were significant SNPs. Four putative candidate genes *BnaC09.RPS6*, *BnaC09.MATE*, *BnaA10.PPD5*, and *BnaC09.Histone* was identified involved in DS tolerance in rapeseed (Shahzad et al., 2021). In a recent study, Varshney et al. (2019) resequenced the whole genome of 429 chickpea lines collected from 45 countries. They identified 122 candidate regions with 204 genes under selection through chickpea breeding. From GWAS analysis, 262 markers and several candidate genes were also identified linked with 13 different traits including DS tolerance (Varshney et al., 2019). This large‐scale study lays the foundation for trait and stress improvement and acceleration of genetic gains in future chickpea breeding programs. For more representative examples, the readers can refer to Table 3.

#### 3.1.2 Transcriptomics

Advances in RNA sequencing technologies using parallel transcriptome profiling offer new insights for analyzing genes and gene networks that respond to DS. Moreover, RNA profiling using microarrays, expressed sequence tags, serial analysis of gene expression, and Affymetrix gene technology have elucidated multiple gene functions under DS (Table 4). In wild‐type and mutant maize plants, transcriptomic profiling identified numerous differentially expressed genes (DEGs) and showed that photosynthesis‐related gene expression was inhibited in wild‐type plants but was mostly unaffected in mutant plants under DS (Zhang, Liu, Wu et al., 2020). An in‐house modified oligonucleotide‐based, drought‐specific microarray system was used to compare tree cotton (*Gossypium arboreum* L.) and upland cotton (*G. hirsutum* L.) root tissue transcriptomic profiles under DS (Ahmad et al., 2020). Of the 500 expression sequence tags identified in upland cotton roots under DS, seven were downregulated, and 256 were upregulated. In contrast, in tree cotton roots, only one expression sequence tag was upregulated, but 325 were downregulated (Ahmad et al., 2020). In a recent study, Singh et al. (2021) identified and validated the expression patterns of NAC genes from three legumes [including chickpea, pigeonpea (*Cajanus cajan* L. Huth), and peanut (*Arachis hypogaea* L.)] under DS conditions. Based on expression analysis, 10 genes from chickpea, six genes from pigeonpea, and five genes from peanut were identified as DS‐responsive candidate genes (Singh et al., 2021). These findings suggest that NAC transcription factors (TFs) play a vital role in conferring DS in many crop plants.

**TABLE 4:** Some examples of omics studies under drought stress conditions in different plant species

Plant specie	Stress condition	Tissue	Approach	Key outcomes	References
**Transcriptomics**					
Maize (*Zea mays* L.)	Water withheld: 8 d	Leaves	RNA‐seq	Identified 4,552 DEGs involved in drought tolerance of wild‐type and mutant; Expression of photosynthesis‐related genes was inhibited in the wild‐type but mostly unaffected in the mutant under DS	Zhang, Liu, Wu et al. (2020)
Wheat (*Triticum aestivum* L.)	Water withheld: 12 and 22 d	Leaves	RNA‐seq	*TaLHB1B2*, *TaLHCA1*, *TaPsbR*, *TaPEX11.B*, *TaPEX11.C*, *TaPEX11.D*, and *TaDRP3A* were downregulated under DS	Sanad et al. (2020)
Rice (*Oryza sativa* L.)	Water withheld; 7 and 14 d	Leaves	BGIseq‐500	743 genes (534 upregulated and 209 downregulated) were significantly differentially expressed between overexpression plants and non‐transgenic plants, which were mainly associated with plant hormone transduction and sugar metabolism; Results elucidated the role of *OsMIOX* in DT	F. Shi et al. (2020)
Safflower (*Carthamus tinctorius* L.)	85, 70, 50, and 30% soil water contents; 5, 9, and 15 d	Leaves	HiSeqTM 2500	Identified 3,280 and 2,260 DEGs as drought‐tolerant and susceptible, respectively, under drought and ample water conditions; Several key candidate genes (e.g., *MYB2*, *MYB62*, *ABA2*, *CYP707A4*, *ZDS*, *GST23*, *GSTL1*, *Cu‐ZnSOD1*, and *ALDH3F1*) were more likely to determine DT in safflower	Wei et al. (2020)
Papaya (*Carica papaya* L.)	Water withheld; 14 d after treatment	Leaves	RNA‐seq	Identified 283 DEGs related to drought; 206 were upregulated, and 61 were downregulated in susceptible plants, while 235 were upregulated and 36 were downregulated in tolerant plants under water deficit vs. optimal watering conditions. The transcription factor‐associated DEGs were classified into 13 major families that are involved in DT mechanisms	Estrella‐Maldonado et al. (2021)
Garden petunia (*Petunia×hybrida* L.)	Water withheld; 5 d	Leaves	RNA‐seq	Gene expression analysis showed that more DEGs were found on day 3 (6417) and day 5 (1233) of water deficit than day 1 (195): 77 DEGs were commonly upregulated, and 69, 2703, and 80 upregulated uniquely on days 1, 3, and 5, respectively. Only 1 DEG was downregulated for all three points; The identified DEGs belong to well‐known transcription factors involved in drought‐related networks	Park et al. (2021)
**Proteomics**					
Rice	Water withheld; 30 d	Leaves	iTRAQ and LC‐MS/MS	Identified 38 differentially upregulated proteins related to DS tolerance, with six (4CLL9, CSLA3, CSLA6, CSLC2, CSLC9, and CSLC10) significantly related to DS tolerance in two accessions	Han et al. (2020)
Common grapevine (*Vitis sylvestris* L.)	Water withheld; 4, 8, 12, and 16 d	Leaves	LC‐MS/MS	Hierarchical clustering of 63 DEPs to detect coordinated regulated proteins in response to DS; Cluster I contained one protein spot (spot 58) involved in stress defense, while Cluster II included 62 protein spots; Comparative proteomic analysis showed that 18 drought‐responsive proteins changed in both accessions under DS, and 48 were variety specific	Azri et al. (2020)
Upland cotton (*Gossypium hirsutum* L.)	40–45% and 70–75% soil relative water contents; 30 and 45 d	Roots	LC‐MS/MS	118 DEPs were upregulated and 105 were downregulated; Identified potential biological pathways and drought‐responsive proteins related to stress or defense responses and plant hormone metabolism under DS	Xiao et al. (2020)
Sorghum [*Sorghum bicolor* (L.) Moench]	Water withheld on V3 stage; 12 d	Roots	iTRAQ	111 DEPs (47%) were significantly upregulated and 126 (53%) were significantly downregulated; Analysis of the root proteome revealed complex protein networks that possibly underpin sorghum responses to water limitation	Goche et al. (2020)
Tobacco (*Nicotiana benthamiana* L.)	30% relative water contents; 5 d	Leaves	iTRAQ	Of 1,087 chloroplast proteins, 329 DEPs were related to metabolic pathways including photosynthesis, photosynthesis‐antenna proteins, glyoxylate and dicarboxylate metabolism, and carbon fixation in photosynthetic organisms	Chen, Li (2021)
Rapeseed (*Brassica napus* L.)	Water withheld; 30, 40, and 50 d	Seeds	HPLC	2,098 proteins identified; 112, 151, and 138 DEPs found at 30, 40, and 50 days, respectively, under DS; Proteome data showed that protein expression increased for fatty acid degradation and protein storage and decreased for fatty acid biosynthesis; Moreover, seed oil contents decreased under DS	Li, Zhang, Hu et al. (2021)
**Metabolomics**					
Cottonwood (*Populus* spp.)	Relative soil moisture content; 50–55% (mild drought), 35–40% (moderate drought) 15–20% (severe drought); 3 wk	Leaves	GC‐TOF/MS	69 and 53 differentially accumulated metabolites identified in drought‐tolerant and sensitive poplar under DS; Carbohydrate, amino acid, lipid, and energy metabolism combined contributed to the common drought responses of two poplar species	Jia et al. (2020)
Earthmoss (*Physcomitrium patens* L.)	Water withheld; 4, 8, and 12 d	Roots	GC‐TOF/MS	30 key metabolites induced by DS; 27 were involved in tricarboxylic acid cycle, glycolysis, starch and sucrose metabolism, GABA shut, and shikimic pathways	Xiong et al. (2020)
Wheat	20% PEG‐6000; 7 d	Leaves	LC‐MS/MS and UHPLC‐MS/MS	Under DS, 691 peaks detected, with 175 identified as known metabolites DT HX10 had higher growth indices than drought‐sensitive YN211; HX10 accumulated a series of phenolics more than YN211; HX10 had almost 13‐fold more thymine, a pyrimidine, than YN211 after DS	Guo, Xin, et al. (2020)
Sorghum	Water withheld; 1 d after every 7 d	Roots and shoots	UHPLC‐HDMS	Significant treatment‐related differential metabolic expression between rhizobacteria‐primed and control plants; Iso‐quinoline alkaloid biosynthesis and glutathione biosynthesis upregulated in plants treated with the selected rhizobacterial isolates. Each isolate had a unique effect on the sorghum metabolome	Carlson et al. (2020)
Maize	Water withheld; 7 d after 12 wk and 7 d after 14 wk	Kernel and inner cob	HRMS	Distinct differentially accumulated metabolites identified under well‐watered conditions, and less divergent differentially accumulated metabolites identified under DS conditions; DS response was dominant over tissue‐specific metabolites	Gaffney et al. (2021)
Tobacco (*Nicotiana benthamiana* L.)	Water withheld; 10 d after 3 wk	Leaves	uHPLC‐DAD, HPLC, GC/MS, and LC‐MS/MS	Metabolic dataset showed increased levels of tryptophan phenylalanine and tyrosine compared with wild‐type; Metabolic changes in transgenic tobacco were less effective for DS adaptation but showed improved tolerance to salt stress.	Oliva et al. (2021)
Peanut (*Arachis hypogaea* L.)	10% PEG; 48 h	Leaves	GC‐MS	Starch, sugars, and polyphenols contents were increased under DS; 160 key metabolites were identified by metabolome analysis; Pinitol, malic acid, and xylopyranose were reported to be stress‐associated metabolites; Identified metabolites were involved in tricarboxylic acid (TCA) and urea cycles; and amino acid biosynthesis	Patel et al. (2022)

*Note*. DEG, differentially expressed gene; DEP, differentially expressed protein; DT, drought tolerance; DS, drought stress; GLM, generalized linear model; MLM, mixed linear model; MTA, marker–trait association; PEG, polyethylene glycol; RNA‐seq, RNA sequencing.

Recently, Wang et al. (2021) identified *GmLHYs* associated genes in soybean under DS using transcriptome profiling. In four pairwise comparisons of transcriptomic data, many DEGs were identified. The wild‐type vs. wild‐type‐drought comparison revealed 8,497 upregulated and 8,917 downregulated DEGs; the quadruple‐mutant vs. quadruple‐mutant‐drought comparison had 9,410 upregulated and 7,591 downregulated DEGs; the wild‐type vs. quadruple‐mutant comparison had 1,263 upregulated and 1,593 downregulated DEGs; the wild‐type with drought treatment vs. *lhy* quadruple‐mutant plants with drought treatment comparison had 2,667 upregulated and 1,071 downregulated DEGs. The four pairwise comparisons revealed that the loss‐of‐function mutant of *GmLHYs* significantly affects soybean drought response mechanisms (Wang et al., 2021). A first‐time comparative transcriptome analysis of lentil (*Lens culinaris* Medik.) under combined heat and DS identified 14,167 DEGs, with 11,724 upregulated and 2,443 downregulated (Hosseini et al., 2021). Moreover, under DS, 1,702 DEGs were identified, with 1,023 upregulated and 679 downregulated, and under heat stress, 4,327 DEGs were identified, with 1,959 upregulated and 2,368 downregulated. The DEGs analysis revealed the upregulation of many TFs, calcium‐dependent protein kinases, cytochrome P450, and antioxidant genes in response to combined heat and drought stress (Hosseini et al., 2021).

#### 3.1.3 Proteomics

Proteomic studies under DS have revealed the functions of various drought‐responsive proteins involved in signal transduction, triggering antioxidant mechanisms, and protecting and acclimatizing plant redox homeostasis (Table 4). A recent evaluation of the DT capacity of 133 weedy rice accessions using phenotypic identification reported that accession WR16 was highly drought tolerant, and proteome analysis (iTRAQ) identified 38 co‐upregulated proteins related to DS tolerance (Han et al., 2020). Moreover, directed parallel reaction monitoring showed that six of nine proteins in weedy rice had positive associations with DS tolerance (Han et al., 2020). The physiological responses of DS have extensively been studied in ginger (*Zingiber officinale* Roscoe), but the molecular mechanisms remain unknown. Differential protein expression under DS revealed 57 upregulated and 41 downregulated proteins relative to the control, which was associated with photosynthesis and phenylalanine metabolism (Lv et al., 2020). Moreover, the protein data analysis advocated that increased cyclic electron flow around photosystem‐I mitigated DS damage at the cost of reduced electron transfer efficiency and photosynthetic rate (Lv et al., 2020).

In potato (*Solanum tuberosum* L.), proteome analysis offered new information on the molecular mechanism of DS tolerance (Zhang, Liu, Qi et al., 2020). Sixteen proteins were differentially expressed with at least twofold abundance between the control and drought treatment. Moreover, the function of differentially expressed proteins in potato leaves were primarily associated with initiating metabolic adjustment processes and activating the defense system under DS (Zhang, Liu, Qi et al., 2020). In wheat, proteomics analysis revealed the mechanisms used by exogenous 5‐aminolevulinic acid to protect against DS; among the 9,499 identified proteins, exogenous 5‐aminolevulinic acid application changed the expression of 469 proteins, whereas under DS the expression of 87 chloroplast proteins was changed (Wang, Li, Liu et al., 2020). The 5‐aminolevulinic acid pretreatment also changed some biological pathways related to photosynthesis and ribosomes to enhance chloroplast DT (Wang, Li, Liu et al., 2020).

Maize seedling responses to mild and severe DS were analyzed using comparative tandem mass tag proteomics and physiological characteristics, which identified 5,601 proteins in maize leaves (Li, Wang, Zhao et al., 2021). Under mild and severe drought, 104 and 464 proteins were differentially expressed, respectively; however, only 30 proteins overlapped. There were more downregulated proteins than upregulated proteins under DS. Severe DS downregulated photosystem‐ and protein synthesis‐related proteins, affecting the photosynthetic network (Li, Wang, Zhao et al., 2021). In another study, proteome analysis was used to annotate the molecular mechanisms underlying the soybean response to water deficit during vegetative stage (Yahoueian et al., 2021). The 488 proteins identified, 20 differentially expressed proteins were implicated in several vital cellular functions, including oxidative stress defense, signal transduction, and photosynthesis, enabling plants to contend with DS using an effective stay‐green mechanism through coordinated gene expression (Yahoueian et al., 2021).

#### 3.1.4 Metabolomics

Recent developments in plant metabolomics studies offer a detailed overview of how plant metabolism responds to various stresses and the underlying complex metabolic regulation (Raza, 2022). Significant accumulation has been reported for various metabolites linked to major cellular metabolic pathways under DS (Table 4). For instance, fulvic acid ameliorates DS‐induced damage in tea plants, but its function during DS is unknown (J. Sun et al., 2020). A liquid chromatography electrospray ionization tandem mass spectrometric study identified 892 metabolites in tea [*Camellia sinensis* (L.) Kuntze] plants treated with fulvic acid under different stages of DS; 54 and 125 differentially accumulated metabolites were identified at two time points (4 and 8 d of DS, respectively). Moreover, fulvic acid enhanced DT by enhancing flavonoid biosynthesis, increasing ascorbate metabolism, and improving glutathione metabolism (J. Sun et al., 2020). A discovery‐based approach with ultra‐performance liquid chromatography–mass spectrometry was used to study various physiological processes related to drought in a metabolic cross profile between drought‐tolerant (HX10) and drought‐sensitive (YN211) wheat genotype under DS (Guo, Xin, et al., 2020). Genotype HX10 had higher growth indices under DS than YN211; its strong DT ability could explain the high accumulation of metabolites, such as phenolics, and high levels of various amino acids, alkaloids, organic acids, and flavonoids (Guo, Xin, et al., 2020).

Recently, Ma et al. (2021) performed nontargeted metabolite profiling using gas chromatography–mass spectrometry to analyze the effect of DS on alfalfa metabolism; they quantified 5,335 unique metabolites of the 151,228 identified spectra, with 3,361 upregulated and 1,794 downregulated. Under DS and controlled conditions, 353 differentially accumulated metabolites were identified, with 25 upregulated and 106 downregulated. Therefore, despite reduced water potential and photosynthesis, the drought‐tolerant cultivar kept growing, indicating that different metabolic pathways contributed to increased drought tolerance (Ma et al., 2021). Another study performed metabolomics analysis of seedling leaves of 55 Turkish *Brachypodium distachyon* (L.) Beauv. accessions using flow infusion electrospray high‐resolution mass spectrometry under the same environmental conditions (Skalska et al., 2021). Three metabolomics groups were identified based on 15, 40, and 75% soil water content. The results revealed that proline levels increased in each group under DS; in addition, sugar and starch, antioxidant synthesis, and polyphenolic metabolism changed, but these changes may have been normal physiological responses to drought including bioenergetic resource provision and adaptations to oxidative stresses (Skalska et al., 2021).

In short, several studies have used omics approaches to provide valuable knowledge on the mechanistic basis of DS tolerance in plants. However, more research using combined omics techniques is needed to explore plant DS tolerance capacity. Notably, integrating transcriptome, proteome, and metabolome data with morphophysiological responses to identify specific adaptive drought responses in plants.

#### 3.1.5 Epigenomics and epigenetics regulations of drought stress

Genome‐wide epigenetic modifications in plants are being stated during development and various stresses, which are frequently associated with gene expression at the transcriptional level. The sum of the biochemical variations in nuclear DNA, posttranslational amendments in histone proteins and differences in the biogenesis of noncoding RNAs in a cell is recognized as an epigenome (Saeed et al., 2022; C. Sun et al., 2021). Epigenetics, an intriguing subject of genetics, mystifies scientists as it meddles with the interaction of DNA with phenotypes (Saeed et al., 2022). According to Arthur Riggs, epigenetics is ‘the study of heritable disruption in gene functions caused by mitotically and meiotically changes that cannot be examined by DNA ‘sequence’ (Varotto et al., 2020). Fundamental mechanisms, such as DNA methylation and histone modification, are key to causing epigenetic changes. Other key processes are chromatin remodeling, and small RNAs also cause heritable changes in the plant (Saeed et al., 2022; C. Sun et al., 2021). Understanding the epigenetic events that happen in a plant while encountering DS could be vital in developing drought‐smart crops. Below we have briefly discussed the role of epigenetic regulation of DS in major agronomic crops.

Water stress affecting maize yield has been discussed previously in several articles. To elucidate the epigenetic response of maize to DS, genome‐wide chromatin data coupled with transcriptomic analysis has been performed (Forestan et al., 2018). Several chromatin‐mediated regulations of gene expression, including noncoding RNAs and the dynamical regulator of histone modification have been discovered (H3K4me3 and H3K9ac) (Forestan et al., 2018). Long noncoding RNA was found in many tissues of a DS maize plant. For instance, Xu et al. (2017) found genes with expressed natural antisense transcripts (NATs), a complex class of regulatory RNAs, in two maize inbred lines carrying multiple loci responsible for DS tolerance, as well as two recombinant inbred lines derived from these two parental lines and fixed for combinations of loci that confer either high or low drought tolerance. Even though the function of NATs in plants is unknown, Xu et al. (2017) discovered 1,769 NAT pairs in two inbred maize lines and two derivative recombinant inbred lines. Interestingly, NATs that are associated with stress response were considerably hypomethylated and had less transposable element sequences than non‐NAT genes. Furthermore, NATs seemed to be abundant in H3K36me3, H3K9ac, and H3K4me3 but not in H3K27me3, indicating an open chromatin configuration at their genomic loci (Xu et al., 2017).

With a total output of 141.75 Tg in 2018, barley is a prominent crop farmed in temperate zones worldwide. The main abiotic factor limiting crop output in barley is terminal DS during grain filling. In caryopsis subjected to terminal drought, stress‐specific 24‐nt heterochromatic small interfering RNA was identified in the promoter regions of the barley cytokinin‐oxidase 2.1 gene (*HvCKX2.1*) (Surdonja et al., 2017). The scientists discovered that the amount of DNA methylation in this gene rose when there was a severe drought. Seeds from the DS mother plant germinated quickly, which was surprising. When barley is subjected to drought and salt stress, multiple distinct methylation sites are induced in leaves compared with roots, as reported in wheat (Chwialkowska et al., 2016). Hemi‐methylations (single CHG or simultaneous CHG and CG asymmetric methylation) were likewise more common in leaves than in roots, although complete methylations (mostly symmetric CG methylation) were more common in roots (Chwialkowska et al., 2016). *HvDRM*, a gene involved in de novo DNA methylation, was downregulated in leaves of DS plants, while its expression was unaffected in the roots (Chwialkowska et al., 2016). The scientists discovered that the methylation level in barley DNA was greater than in other crops including rapeseed, rice, and maize. This might be due to the barley genome's large number of repetitive sequences (Mascher et al., 2017). This effect has also been shown in angiosperms, where genome‐wide DNA methylation levels have been linked to the growth of repetitive elements (Niederhuth et al., 2016). On a chromatin level, DS barley plants had denser nucleosome packing, and *HSP17* was identified as one of the drought‐responsive genes (Temel et al., 2017).

The two lines (drought sensitive and drought tolerant) of fava bean (*Vicia faba* L.) were subjected to DS to examine the methylation rate. Under DS, a higher demethylation rate was observed in the tolerant line than in the sensitive (Abid et al., 2017). Furthermore, the demethylation also triggered the expression of genes responsive to DS. Traditional approaches and high‐throughput microRNA deep sequencing have been used to identify drought‐responsive microRNAs in many legume species (Mantri et al., 2013). In another study, Khandal et al. (2017) used deep sequencing to identify 259 microRNAs that were differentially expressed in chickpea root apex during drought and salt stress. Some of these genes are expressed in the same way in salt‐treated barrel clover (*Medicago truncatula* Gaertn.) and soybean root tips, whereas others have distinct expression patterns. Many of them include auxin‐ and abiotic stress‐responsive *cis*‐elements in their promoters, suggesting that phytohormone accumulation controls their regulation. MiR408 transcripts were accumulated in chickpea during drought stress according to Hajyzadeh et al. (2015). These examples suggest that epigenetics or epigenomics play a vital role in understanding the DS responses and tolerance mechanisms at epigenetics level.

### 3.2 Transgenic approaches for drought management

#### 3.2.1 Transgenic plants

Transgenic strategies have been used extensively in the past couple of decades to strengthen plant growth and development under water‐limited conditions. Table 5 shows the details of recent transgenic studies to improve plant function under DS. Extreme environmental conditions such as DS affect rice production and quality worldwide. The function of receptor‐like kinases in plant development is well known, but little is known about the role of S‐domain receptor‐like kinases in controlling root growth. Recently, S‐domain receptor‐like kinase *OsESG1* was identified in rice and recognized for its contribution to early crown root development and drought response (Pan et al., 2020). The *OsESG1* mutant rice plants had fewer crown roots and shorter shoots than the wild type. Moreover, auxin signaling and polar auxin transport were disrupted (Pan et al., 2020). Similarly, *OsNADK1* is a cytosol‐localized NADK gene in rice; the *OsNADK1* mutant had dwarf phenotype at the heading stage, escalating the DS sensitivity and oxidation states in rice cells, indicating that an intracellular redox balance mediated by *OsNADK1* is involved in the drought tolerance of rice (Wang, Li, Ma et al., 2020).

**TABLE 5:** Some examples of transgenic studies under drought stress conditions in different plant species

Plant specie	Stress condition	Gene name	Key outcomes	References
Rice (*Oryza sativa* L.)	15% PEG; 5 d	*OsESG1*	Modulates DS response by regulating antioxidative activity and stress‐related gene expression	Pan et al. (2020)
Rice	Water withheld; 10 and 14 d	*OsNADK1*	Decreased NADP(H)/NAD(H), ascorbic acid (ASA)/dehydroascorbate (DHA), and reduced glutathione (GSH)/oxidized glutathione (GSSG) ratios, increased oxidation states, and sensitivity to DS	Wang, Li, Ma et al. (2020)
Rice	Water withheld; 21 d	*OsRab16A*, and *AtDREB1A*	Improved relative leaf water content, osmoprotectant proline accumulation, and catalase activity, and decreased H_2_O_2_ accumulation	Ganguly et al. (2020)
Rice	Water withheld; 21 d	*OsTZF5*	Overexpression of *OsTZF5* under the control of rice stress‐responsive *OsNAC6* promoter conferred DS	Selvaraj et al. (2020)
Rapeseed (*Brassica napus* L.)	Water withheld; 7 d	*BnKCS1‐1*, *BnKCS1‐2*, and *BnCER1‐2*	High‐density wax crystals on the leaf surface increased the level of aldehydes, alkanes, secondary alcohols, and significantly reduced ketone level	Wang, Jin, Xu et al. (2020)
Sugarcane (*Saccharum officinarum* L.)	Water withheld; 15 d	*Gly III*	Decreased oxidative damage caused by ROS, high relative water contents, chlorophyll, photosynthesis rate, gaseous exchange, and proline contents	Mohanan et al. (2020)
Potato (*Solanum tuberosum* L.)	20% PEG‐6000; 3, 6, 12, 24 h	*StRFP2*	Drought‐stressed transgenic potato plants had significantly higher *StRFP2* expression than the non‐transgenic plants (WT); Moreover, transgenic plants had higher free proline content and catalase activity than the WT	Qi et al. (2020)
Eastern cottonwood (*Populus deltoides* L.)	70% relative soil water contents; 20 d	*PdC3H17*	Overexpression of *PdC3H17* conferred drought tolerance by maintaining high stem water potential, and increased photosynthetic and ROS‐scavenging abilities, enhancing tolerance to DS, compared with controls	Zhuang et al. (2020)
Rice	Water withheld; 10–14 d after 4 weeks, 6 μM abscisic acid	*OsMFT1*	*OsMFT1* acts as a key regulator and plays a vital role in the DS response of rice; it also directly interacts with *OsbZIP66* and *OsMYB26* in the nucleus to regulate transcriptional ability to mediate drought‐related gene expression	Chen, Shen, et al. (2021)
Rice	Water withheld; 21 d after 4 wk	*OsADR3*	Overexpression of *OsADR3* in rice increased DS tolerance ability by enhancing ROS‐scavenging ability and abscisic acid sensitivity	Li, Zhang, Yang et al. (2021)

*Note*. DS, drought stress; PEG, polyethylene glycol; ROS, reactive oxygen species.

The demand for aromatic rice varieties with distinct fragrances is very high in domestic and international markets. However, their yields are significantly affected by biotic and abiotic stresses. Transgenic aromatic rice variety (Pusa Sugandhi 2), which independently overexpressed *AtDREB1A* and *OsRab16A* genes, displayed improved tolerance to drought conditions and was associated with enhanced relative leaf water content, reduced inhibition of shoot and root lengths, reduced H_2_O_2_ accumulation, and increased accumulation of proline and CAT compared with the wild‐type plants (Ganguly et al., 2020). Increased amounts of cuticular wax in plants have been associated with enhanced tolerance to abiotic stresses. Transgenic rapeseed plants overexpressing *BnKCS1‐1* (ortholog of 3‐ketoacyl‐CoA synthase), *BnKCS1‐2*, and *BnCER1‐2* (orthologs of ECERIFERUM) had significantly more cuticular wax than wild‐type plants and showed increased DS tolerance because of reduced water loss (Wang, Jin, Xu et al., 2020). Scanning electron microscopy revealed that overexpression of these genes in transgenic plants resulted in a higher density of leaf surface wax crystals than the wild‐type plants (Wang, Jin, Xu et al., 2020).

These exceptional achievements in transgenic research have improved physiological and molecular traits related to DS; increased antioxidant enzyme activities, proline level, soluble sugar content, photosynthesis rate, transpiration rate, and stomatal conductance; and reduced lipid peroxidation, thus encouraging researchers to develop next‐generation drought‐smart transgenic plants. Various transgenic plants have been produced by expressing DS‐related transgenes; however, engineering genes in the same cultivars could be more prolific as tolerance to DS horizontally transferred in plants.

#### 3.2.2 CRISPR/Cas system: A promising genome editing toolkit

**FIGURE 4:**
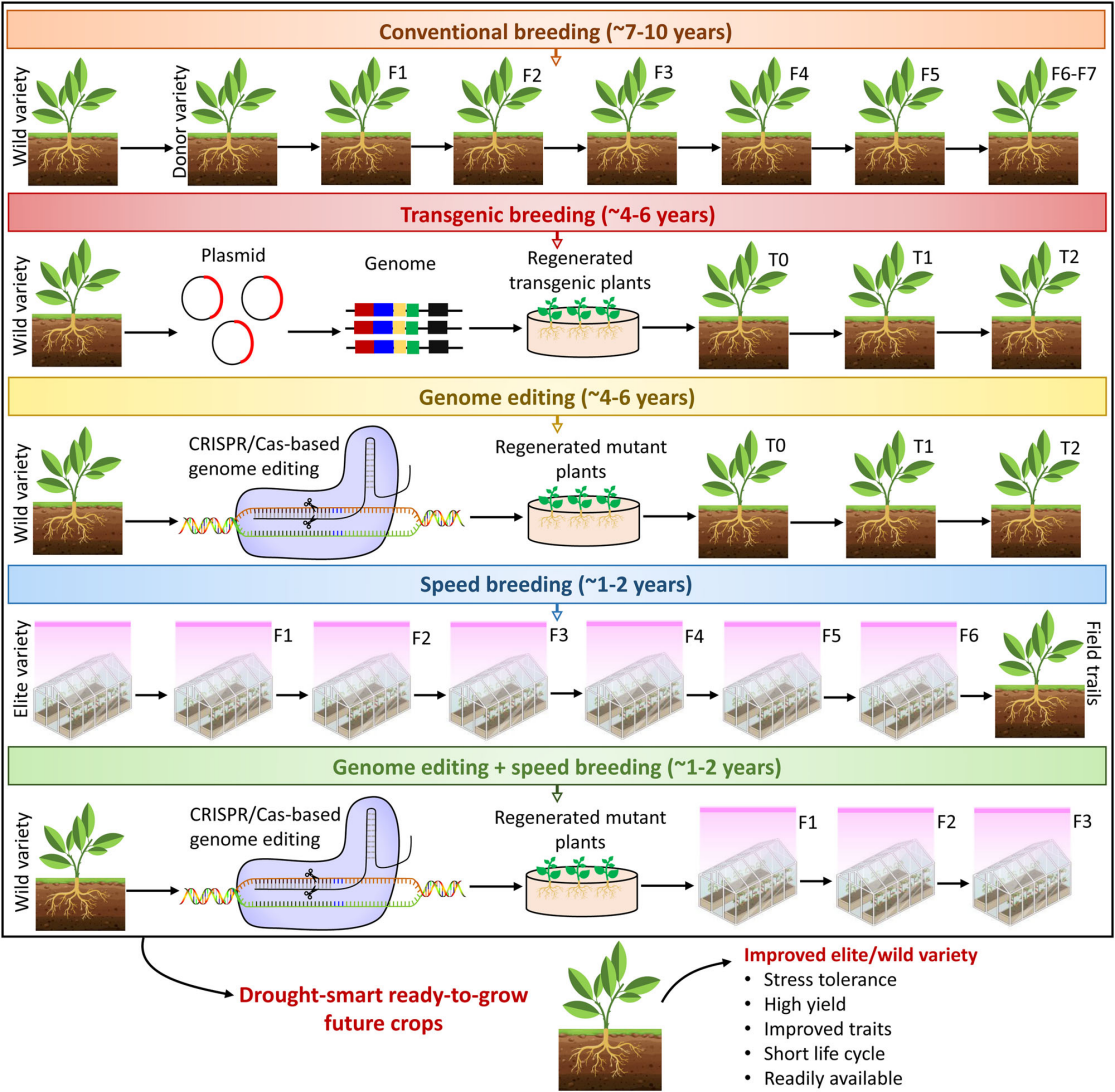
A graphic representation shows the differences between conventional breeding, transgenic breeding, genome editing (CRISPR/Cas system), and speed breeding applications for crop advancement under controlled or stressed conditions. Ultimately, the combination of genome editing and speed breeding can help develop the drought‐smart, ready‐to‐grow future crops in a short time to meet the world food supply

Conventional breeding and transgenic methods have improved DS tolerance in many crop species (Figures 3 and 4). However, most of these promising cultivars cannot produce high yields under water stress. Thus, there is potential for new interventions to diminish the negative effects of DS (Joshi et al., 2020). Newly emerged gene‐editing tools, such as the state‐of‐the‐art Clustered Regularly Interspaced Short Palindromic Repeats (CRISPR) and CRISPR‐associated‐Cas proteins (CRISPR‐Cas9) systems, are preferred by researchers for editing plant genomes for tolerance against a variety of abiotic stresses including DS (Shinwari et al., 2020). Many studies have revealed the important role of TFs and their corresponding resistance genes (*R* genes) for regulating responses against DS. However, little attention has been given to susceptible genes (*S* genes) and their corresponding *trans*‐acting elements that negatively regulate DS tolerance in plants (Zafar et al., 2020). Therefore, disrupting *S* genes or negative regulators of DS‐responsive pathways in plants using CRISPR‐Cas9 could show adaptive resilience to water scarcity.

Few studies have used CRISPR‐Cas9 technology to develop DS‐tolerant plants. Under the ever‐growing climate change, engineering crop plants that tolerate abiotic stresses is essential for sustainable crop production. A drought‐ and salt‐tolerance gene was recently mutated in an *indica* mega rice cultivar “MTU1010” using the CRISPR‐Cas9 system. The mutant plant had wide leaves and decreased stomatal activity, enhancing leaf water preservation under DS. The decreased stomatal opening in *dst* mutant rice plants was attributed to stomatal gene downregulation (Kumar et al., 2020). In *Arabidopsis* and cotton, overexpression of the *HB12* gene decreased ABA sensitivity and salt and drought tolerance by suppressing the expression of ABA‐responsive and stress‐related genes such as *DREB2A*, *RD22*, *RD28*, *SOS2*, *HKT1*, and *SOS2* (He et al., 2020). Suppression of *GhHB12* increased abiotic stress tolerance in cotton (He et al., 2020). Therefore, CRISPR‐Cas9 mediated simultaneous editing of *GhHB12*, and homologous genes were used to develop early maturing cotton cultivars with enhanced adaptability to changing environmental conditions (He et al., 2020).

A WRKY TF of sea island (or pima) cotton (*G. barbadense* L.) (*GbWRKY1*) was previously identified in cotton as a defense‐related gene it can activate the expression of *JAZ1*, which negatively regulates the plant response to fungal pathogens (Luo et al., 2020). *GbWRKY1* overexpression in transgenic *Arabidopsis* and cotton increased drought and salt sensitivity (Luo et al., 2020). Therefore, it is evident that *GbWRKY1* acts as a negative regulator of drought and salt tolerance. Similarly, in wheat, R2R3‐type MYB TFs contributed to multiple abiotic stress responses (Li, Tang, Li et al., 2020). In rice, rolled leaf mutant plants were acquired by mutating *SLR1* and *SLR2* genes using the CRISPR‐Cas9 system (Liao et al., 2019). Mutant plants had decreased stomatal conductance and transpiration rates and increased panicle numbers. Moreover, homozygous mutant plants had higher survival rates, antioxidant (SOD and CAT) activities, and ABA contents, and lower MDA contents compared with its wild type. These results reveal the fundamental value of genome editing, exploring new avenues of leaf rolling protein networks, and DS tolerance in rice (Liao et al., 2019).

In addition to *R* gene overexpression, *S* genes or negative regulators (e.g., *GhHB12*, *GbWRKY1*, R2R3‐type *MYB* genes) of DS tolerance mechanisms could serve as potential target sites for DS tolerance (Table 6). For example, maize gene *ARGOS8* (a negative regulator of ethylene responses) was edited using CRISPR‐Cas9 system to create ubiquitous and improve expression levels in various developmental stages. The results showed that the *ARGOS8* variants increased grain yield by 336.255 kg ha^−1^ (5 bushels acre^−1^) under flowering stress conditions and had no yield loss under DS conditions in the field environment (J. Shi et al., 2017). Furthermore, CRISPR‐Cas9 system variants have been used to engineer drought tolerance through targeted modulation of key *S* and *R* genes in multiple crop plants. Thus, CRISPR tools offer diverse applications for mitigating the effect of DS through multiplex gene editing, base editing, and prime editing.

**TABLE 6:** Examples of CRISPR/Cas‐mediated drought stress tolerance in different plant species

Plant specie	Stress condition	Gene name	Modification	Key outcomes	Reference
Rice (*Oryza sativa* L.)	10% PEG‐6000; 15 d	*Slwrky81*	Knock out	*SRL1* and *SRL2* loss‐of‐function mutations led to a rolled leaf phenotype with drought tolerance; Mutant plants had decreased stomatal conductance and transpiration rate	Liao et al. (2019)
Rice	20% PEG‐6000; 6 d	*Osdst*	Knock out	Mutant plant had enhanced leaf width, reduced stomatal density, and enhanced stomatal closure through modulating H_2_O_2_ homeostasis; Cas9‐free *dst* D184‐305 mutant had moderate tolerance to osmotic stress at the seedling stage	Kumar et al. (2020)
Tomato (*Solanum lycopersicum* L.)	10% PEG‐6000; 1, 3, 6 h	*Sllbd40*	Knock out	Knock‐out plant had improved water‐holding ability under DS, as evidenced by water loss rate and midday leaf water potential	Liu, Li, Li et al. (2020)
Sea Island Cotton (*Gossypium barbadense* L.)	40% PEG‐8000; 8 d	*Gbwrky1*	Knock out	*Gbwrky1* acts as a negative regulator of abscisic acid signaling through an interaction network involving JAZ1 and ABI1 to regulate salt and drought tolerance. *Gbwrky1* mutant plants had enhanced tolerance to DS by regulating abscisic acid signaling	Luo et al. (2020)
Wheat (*Triticum aestivum* L.)	7.5% PEG‐6000; 2 h	*Tampc1‐d4*	Knock out	Mutant plants had increased relative water content and antioxidant enzyme activities and activated some stress‐related and antioxidant‐related genes to mitigate DS	Li, Tang, Li et al. (2020)
Upland cotton (*Gossypium hirsutum* L.)	15% PEG‐6000; 1, 3, 6, 12, 24 h	*GhHB12*	Knock out	Downregulation of *GhHB12* increased tolerance to multiple abiotic stresses in cotton	He et al. (2020)
Soybean (*Glycine max* L.)	7%, 8%, 9% PEG‐8000; 1, 3, 6, 12, 24 h	gma‐miR398c	Knock out	*Gma‐miR398c* overexpression increased relative electrolyte leakage and stomatal opening because of the elimination of ROS‐scavenging ability; Downregulation of the *gma‐miR398c* gene improved tolerance to DS by decreasing relative electrolyte leakage and stomatal opening	Zhou et al. (2020)
Soybean [Glycine max (L.) Merr.]	Water withheld; 10 d	*LHY1a*, *LHY1b*, *LHY2a*, *LHY2b*	Knock out	*GmLHYs*, the core clock components, negatively control drought tolerance in soybean; The quadruple mutants of *GmLHYs* had reduced water loss rates under drought, and thus enhanced tolerance to drought through abscisic acid regulation	Wang, Bu, Cheng et al. (2021)

### 3.3 Conventional and modern breeding platforms for drought management

#### 3.3.1 Conventional breeding

Conventional breeding identifies parental lines with desirable traits to generate a favorable combination of the new line for the next generation (Acquaah, 2015). Conventional breeding has been occurring for the last 10 decades and has played a vital role in people's need for food, feed, and fiber (Doebley et al., 2006; Varshney et al., 2021c). In the early days of crop farming, farmers selected the wild relatives of already domesticated crops with extant superior variation and enhanced immunity to unfavorable conditions. Cashing the early efforts made by breeders, farmers have had significant success in the last couple of centuries, generating varieties with high yield and good nutritional quality (Kaiser et al., 2020).

Farmers faced abiotic stress constraints such as DS in the early days (and now), with some regions experiencing severe and prolonged DS (Cheng et al., 2021). Some researchers experimented by subjecting cultivars released in different years to water‐scarce and well‐watered conditions (Araus et al., 2002; Tollenaar & Lee, 2002). For example, several sorghum [*Sorghum bicolor* (L.) Moench] cultivars subjected to DS at the reproductive stage showed tolerance to DS (Akman et al., 2021). A total of 65 genotypes of durum wheat [*Triticum turgidum* L. subsp. *durum* (Desf.) van Slageren] were tested under different water regimes, and environments generally outclass the earlier released cultivars because of their better adaptability and higher yield (De Vita et al., 2010).

A case study for barley conducted across Europe revealed that the tested population of genotype exhibited less genome × environment interaction with enhanced yield under DS (Rizza et al., 2004). Similarly, sugar beet (*Beta vulgaris* L. subsp. *vulgaris*) genotypes subjected to a series of drought‐prone and well‐watered conditions displayed identical outcomes (Ober et al., 2004). Comparing a large set of cultivars makes identifying the best ones with desirable traits under water‐scarce conditions easier than using a small set. However, breeding for drought tolerance mainly depends on the yield potential of parental lines rather than tolerance‐related traits (Mastrangelo et al., 2012). Potato genotypes were subjected to DS in multiple environments and days to permanent wilting point used to screen for drought tolerance (Kivuva et al., 2015). The study identified a series of genotypes with enhanced DS tolerance based on high root dry mass and days to permanent wilting point (Kivuva et al., 2015). The grain development stage is important for determining overall crop yield, but DS at this stage can reduce grain yield manifold (Torres & Henry, 2018). Even mild DS at flowering can severely impact rice grain yield (Torres & Henry, 2018). Torres & Henry (2018) examined the response of a set of rice breeding lines to varying levels of DS at flowering; Binuhangin and IR70215‐70‐CPA‐3‐4‐1‐3 showed excellent tolerance to mild and moderate DS. Interestingly, there was no correlation between the genotypes for stomatal conductance rate or root dry mass with grain or total dry matter yield (Torres & Henry, 2018). Another study—at the CIMMYT Norman E. Borlaug Research Station—evaluated the performance of 30 wheat cultivars released in the past 50 yr (Mondal et al., 2020), yielding significant results as a set of cultivars (24 bread wheat and six durum wheat) showed relatively good performance under prolonged DS. Thousand‐grain weight was associated with DS tolerance, an important parameter to determine productivity, which could be crucial for future studies (Mondal et al., 2020).

In recent times, conventional breeding has been surpassed by molecular breeding, speeding up cultivar development. However, conventional breeding remains important, as it produces transgene‐free crops with better nutritional value and higher uniformity in yield‐related traits (Ahmar et al., 2020). In addition, 95% of organic produces comes directly from the conventional breeding sector (Van Bueren et al., 2011). Some DT crop varieties from around the world are in Supplemental Table S1.

#### 3.3.2 Speed breeding: A time‐saving method for advancing generations

Conventional breeding generally takes 8–10 yr to generate a new variety (Figure 4). The growing world population has increased the food security risk and needs advanced approaches to maximize crop yields. Figure 4 shows the differences between several breeding options to develop stress‐smart crops. Rapid generation advance, or ‘speed breeding,’ shortens the overall growth cycle and speeds up the breeding process (Gaur et al., 2007; Bhattarai et al., 2009; Saxena et al., 2019; Fikre et al., 2021) that involves the production of miniature plants from immature seeds in a controlled environment. After producing a few flowers, the seeds are then collected for the next sowing cycle. Speed breeding is useful for developing molecular markers because the most limiting factor in marker development is the seed‐to‐seed breeding cycle (Ochatt et al., 2002; Ochatt & Sangwan, 2008; Saxena et al., 2019; Fikre et al., 2021). Speed breeding reduces the duration of variety development and increases overall crop production (Figure 4) (Watson et al., 2018). A recent example of speed breeding produced seven generations of chickpea per year, which could be pivotal in curbing food security threats (Samineni et al., 2020). Likewise, Fikre et al. (2021) has obtained four generations of working chickpea seeds (F_2_–F_5_) using two research locations to improve the DS and yield in commercial cultivars. They reported that the average time essential to acquire early matured pods differed from 80 to 85 d. Notably, harvesting four generations in an annual cycle enables savings of ∼50% time in variety release, which can accelerate the rate of genetic gain in new varieties (Fikre et al., 2021). Recently, Saxena et al. (2019) proposed that speed breeding can be potentially used to accelerate the genetic gain in pigeonpea and to develop rapid generations. Habitually, this legume has been a photoperiod‐sensitive crop that requires prolonged intervals of darkness to induce flowering.

Speed breeding can be used to develop drought‐smart future cultivars (Watson et al., 2018). A research group in Germany used specialized LED lights to shorten crop daylength to develop fast‐breed cultivars for short‐day crops such as rice (Jähne et al., 2020). Rice plants were exposed to a blue‐light‐enriched and far‐red‐deprived light spectrum; the blue light shortened the time to flowering (Ahmar et al., 2020; Jähne et al., 2020), revealing the role of light quality in speed breeding. An Australian study used speed breeding methodology to develop a wheat variety that can survive water‐scarce conditions (Christopher et al., 2015); the group produced up to F_5_ generation of stay‐green inbred lines within 18 mo. The research also yielded more than 40,000 molecular markers for identifying novel QTL responsible for stay‐green traits (Christopher et al., 2015). However, no other research is available concerning speed breeding for DS‐tolerant crop plants. Therefore, more impetus is required in this area to minimize the risk of DS by developing advanced generations in a short time.

### 3.4 Biochemical and mechanical options for drought management

#### 3.4.1 Phytohormone applications

Phytohormones, including auxins, gibberellic acid, cytokines, ABA, ethylene, jasmonic acid (JA), SA, strigolactones, and brassinosteroids (Hafeez et al., 2021; Mubarik et al., 2021; Raza et al., 2022), are signal molecules and essential components for regulating plant growth under DS (Table 7) (Mubarik et al., 2021). Foliar application of auxin (auxin or indole‐3‐acetic acid 3,000 mg L^−1^) to sugarcane at the mid‐flowering stage increased grain yield, head number, 1000‐grain weight, and soluble carbohydrates of the safflower (*Carthamus tinctorius* L.) cultivar Goldasht under DS (Mousavi et al., 2022). Applying cytokines (10 mg L^−1^ 6‐benzylaminopurine) to wheat cultivars enhanced photosynthetic content, growth, and plant stability under DS (Kumari et al., 2018). Elevated endogenous ABA production enhances plant turgor pressure that ultimately increases DS tolerance (Vishwakarma et al., 2017). In *Arabidopsis*, ABA signaling under DS reduced photosynthetic activity by closing stomata that ultimately reduced water loss mainly by regulating the transcript level of *HOS15* gene in an ABA‐dependent manner (Ali & Yun, 2020). Foliar application of ABA in wheat enhanced plant photosynthesis and increased seed yield (Dwivedi et al., 2018).

**TABLE 7:** Summary of experiments indicating the positive role of phytohormones in mitigating drought stress in several plant species

Plant specie	Stress condition	Form and dose of hormone	Key findings and protective role	References
Tobacco (*Nicotiana tabacum* L.)	15% PEG‐6000; 14 d	0.3 mmol L^−1^ SA	SA enhanced SOD, catalase, and POD activities, proline, and soluble protein, and decreased MDA activity; SA improved pigment biosynthesis and photosystem repair under DS by enhancing photosynthesis	Feng et al. (2021)
Persian petunia *(Petunia* sp.)	50, 25% field capacity; till harvest	0, 100, 200, 300 mg L^−1^ SA and GA3	SA increased proline content and root to shoot ratio and decreased stomatal conductance in stressed plants	Goldani et al. (2021)
Sweet basil (*Ocimum basilicum* L.)	60% field capacity; 0.01 mM Tween‐20, 14 d	30 mM trehalose; 1 mM SA	Reduced oxidative stress, enhanced photosynthesis and plant growth, and increased SOD, POD, catalase, proline, and glycine betaine	Zulfiqar et al. (2021)
Rice (*Oryza sativa* L.)	15% PEG‐6000; 7 d	1 mmol L^−1^ SA	Increased antioxidant levels (guaiacol peroxidase, APX, catalase, proline, shoot weight and length; decreased MDA and H_2_O_2_ contents	Sohag et al. (2020)
Tropical carpet grass (*Axonopus compressus* L.)	40% field capacity; 14 d	100 μmol ABA	Exogenous application enhanced SOD, APX, and POD activities, phenolics, proline, proteins, sugars, and chlorophyll content and decreased H_2_O_2_ and MDA contents	Nawaz & Wang (2020)
Pearl millet (*Pennisetum glaucum* L.)	20% PEG‐6000; 7 d, 14 d	100 μM JA, 100 μM ABA	JA and ABA enhanced DT by increasing antioxidant activities, chlorophyll, and relative water content	Awan et al. (2021)
Maize (*Zea mays* L.)	Water deficient; 5 d	20 μM MeJA	Reduced oxidative damage because of drought stress by decreasing MDA, H_2_O_2_, and lipoxygenase activity; Increased POD, SOD, CAT, proline, carbohydrate, total sugars	Tayyab et al. (2020)
Peanut (*Arachis hypogaea* L.)	40% soil relative water content, 7 d, 14 d	0, 0.05, 0.10, 0.15, 0.20 ppm BR	Optimum priming with 0.15 ppm BR reduces drought inhibition and enhance yield; Gene ontologies and metabolic pathways enriched differentially expressed gene produced with BR priming+drought; BR priming rescue the optimized level of auxin and gibberellin under DS	Huang et al. (2020)
Wheat (*Triticum aestivum* L.)	50% field capacity; till harvest	0.5 μM 24‐epibrassinolide	Enhanced crop yield and improved activities of glycine betaine, total sugars, amino acid, proline, and antioxidants (APX, glutathione reductase, POD, SOD, catalase)	Zeid et al. (2019)
Upland cotton (*Gossypium hirsutum* L.)	60% field capacity, 9 d	2, 4, 6, 8 mM acetic acid; 100 μM JA; 12 μM ABA	Increased chlorophyll content and photosynthesis in roots after 6 and 9 days of treatment	Li, Kong, Luo et al. (2021)
Common wheat (*Triticum sativum* L.)	−1.03 MPa PEG‐6000; 16 d	0.1 mM JA, 0.5 mM kinetin	Elevated levels of antioxidants (APX, SOD, POD, catalase), shoot to root ratio, proline, chlorophyll content, and soluble carbohydrates	Abeed et al. (2021)

*Note*. APX, ascorbate peroxidase; BR, brassinosteroids; DT, drought tolerance; DS, drought stress; JA, jasmonic acid; MDA, malondialdehyde; PEG, polyethylene glycol; POD, peroxidase; SA, salicylic acid; SOD, superoxide dismutase.

Applying JA (0.5–10 μM) enhanced DS tolerance in sugar beet by improving antioxidant activities and consequently increasing plant yield (Ghaffari et al., 2019). Exogenous application of acetic acid (8 mM), JA (100 μM), and ABA (12 μM) enhanced DS tolerance in cotton by improving photosynthesis and chlorophyll content. Acetic acid increased the expression of ABA genes (*NCED2*, *NCED3*, and *NCED9*) in leaves and JA genes (*GhAOS6*, *GhLOX3*, and *GhOPR11*), increasing cotton survival under DS by decreasing transpiration rate and stomatal conductance (Li, Kong, Luo et al., 2021). Two turnip (*Brassica rapa* L.) genotypes (KS101 and KBS3) treated with epibrassinolide (EBL, 0.01 μM) and JA (10 μM) under DS had enhanced antioxidant activities (ascorbate peroxidase, CAT, POD, glutathione reductase, and SOD), transpiration rate, stomatal conductance, and photosynthetic rate (Lone et al., 2021). Applying EBL (24‐epibrassinolide) to purple coneflower [*Echinacea purpurea* (L.) Moench] under polyethylene glycol‐induced DS enhanced antioxidant activities (SOD, CAT, POD), proline content, and total proteins and decreased MDA and H_2_O_2_ levels (Hosseinpour et al., 2020).

Applying SA (600 μM) and K (69 mg 5 kg^−1^) to spinach (*Spinacia oleracea* L.) under DS enhanced Chl content (88.4%), plant dry weight (58%), root length (44.5%), and shoot length (33%) (Gilani et al., 2020). Foliar application of SA to pistachio (*Pistacia vera* L.) improved pigment contents, Chl, carotenoids, osmolyte accumulation, and antioxidant defense activity (Haghighi et al., 2021). Applying naphthalene acetic acid (20 and 40 ppm) and SA (50 and 100 ppm) to tomato (*Solanum esculentum* Mill.) under 20% soil moisture reduction at 50% flowering enhanced RWC, Chl index, membrane stability, and Chl stability index (Mumithra Kamatchi et al., 2020). See Table 7 for more key examples on the protective role of phytohormones against the adverse effects of DS in different plant species.

##### 3.4.1.1 Agronomic practices

Agronomic practices, such as water management, adjusting plant density, and nutrient management, are the backbone for increasing crop production under seasonal DS. Other strategies include zero tillage, mulching, intercropping, and deep plowing (Mupangwa et al., 2007; Johnson et al., 2018; Chai et al., 2021). Applying gypsum is important for soils with low infiltration capacity (Hamza & Anderson, 2003). Integrating mung bean [*Vigna radiata* (L.) R. Wilczek var. *radiata*] residue in a maize–wheat cropping system significantly increased WUE and yield (Jat et al., 2018), which could enhance crop productivity in water‐scarce conditions. Other strategies, such as nutrient management and mulching, to reduce the adverse effects of DS are discussed below.

##### Plant density and time of sowing

Sowing date is important for mitigating the devastating effects of DS at the reproductive stage (Turner, 2004). However, this strategy largely depends on crop type. Early sown maize avoided the potential drought and high temperatures in mid‐summer, significantly increasing maize growth (Lu et al., 2017). Similar results were reported for early sown soybean in Serbia, with late sowing exposing the plants to less precipitation and potential DS (Mandić et al., 2020). Wheat genotypes sown at Faisalabad (Pakistan) during December 2016 successfully mitigated drought and high temperature (Ihsan et al., 2016). The genotypes sown in December produced more tillers and had a longer grain‐filling duration with increased overall yield than the one sown in January (Ihsan et al., 2016).

Some studies have reported that increasing plant density can uphold the remaining soil by shading the soil surface. This water can be used by plants during later growth stages. For example, high plant density of maize crop decreased leaf area index, thus reducing evapotranspiration and enhancing WUE under arid conditions (Guo et al., 2021). However, different legume plants grown at high density had low yields and increased lodging under DS (Nadeem et al., 2019). Studies are needed to examine the effects of high and low planting density and sowing time in various drought‐prone locations.

##### 3.4.1.2 Nutrient management and mulching

Excessive N fertilizer use contaminates groundwater because of N leaching. A ridge–furrow strategy that incorporated ridge mulching reduced N leaching and enhanced WUE and crop yield on China's Loess Plateau (Liu et al., 2017). In another study, the use of ridge–furrows and plastic mulching with higher N levels augmented the soil's water retention capacity, increased N use efficiency, and boosted rainfed maize grain yield by 70% compared with lowered N levels (Gang et al., 2019). Film mulching could be vital for increasing water storage in dryland areas. Year‐round film mulch enhanced water storage in 2‐m‐deep soil under winter wheat grown on the Loess Plateau, improving grain yield and minimizing soil N leaching and water‐holding capacity (Li, Xie, Gao et al., 2019). The role of K has been implicated in DS tolerance by improving WUE (Hassan et al., 2017; Jákli et al., 2016). Mulching of various other crops, particularly for WUE, can reduce the adverse effects of DS on plant growth and development (Qin et al., 2015, 2021). For example, in winter wheat under semi‐arid conditions, straw strip mulching significantly reduced soil temperature relative to plastic film mulching and non‐mulching, thus reducing evaporation and ultimately increasing water‐holding capacity (Li, Chai, Chai et al., 2021). Soil K application improved WUE, leaf K content, leaf water potential, and gaseous exchange in eucalyptus (*Eucalyptus urophylla* S. T. Blake) (Santos et al., 2020). Potassium is a potential regulator of DS tolerance, particularly in plants grown in low‐K soil (Santos et al., 2020).

## 4 CONCLUDING REMARKS AND FUTURE OUTLOOK

Climate change, food and water scarcity, and the growing population are issues being encountered worldwide. Drought stress has a massive effect on crop growth and productivity and is counteracting the ‘zero hunger’ goal, with its strength and severity anticipated to increase in upcoming years. Plant tolerance to DS depends on the impact, duration, and intensity of the stress and plant development stage. This review covered various plant responses to DS and the associated tolerance mechanisms. Plants respond to DS via morphological, physiological, biochemical, and molecular mechanisms that vary between species.

Significant progress has been made to improve DS tolerance by adapting conventional to modern breeding and biotechnological tools. Traditional breeding has helped develop new cultivars; however, there was an urgent need to develop time‐saving methods because of the increased global food demand. Speed breeding emerged as a method for rapidly developing climate‐smart cultivars (Figure 4). Advances in next‐generation breeding techniques and biochemical processes have modernized crop breeding. As a result, combining a variety of traditional and modern biotechnological techniques, such as genomics (QTL mapping, GWAS, and genomic selection), transcriptomics, metabolomics, proteomics, epigenomics, and genome editing (CRISP/Cas system), transgenic approaches, biochemical methods (seed priming or exogenous treatment with phytohormones), and agronomic practices, will significantly improve our current understanding of DS responses and tolerance mechanisms in crop plants. Identifying new key genes and QTL, metabolites, and proteins related to DS‐responsive mechanisms can be a potential candidate for CRISPR/Cas‐mediated genome editing, combined with speed breeding, could be used to develop new DS‐tolerant cultivars to achieve ‘zero hunger’ goal and feed the growing population (Figure 4). Information from several scientific fields, including plant genetics, plant physiology, and plant biochemistry with omics tools and modern speed breeding, is needed for plant scientists to balance their investigations in crop improvement under harsh environmental conditions including DS.

## AUTHOR CONTRIBUTIONS

Ali Raza: Conceptualization; Investigation; Writing‐original draft; Writing‐review & editing. Muhammad Salman Mubarik: Investigation; Writing‐original draft. Rahat Sharif: Investigation; Writing‐original draft. Madiha Habib: Investigation; Writing‐original draft. Warda Jabeen: Investigation; Writing‐original draft. Chong Zhang: Writing‐review & editing. Hua Chen: Writing‐review & editing. Zhong‐Hua Chen: Writing‐review & editing. Kadambot H. M. Siddique: Writing‐review & editing. Weijian Zhuang: Funding acquisition; Resources; Supervision; Writing‐review & editing. Rajeev K. Varshney: Funding acquisition; Resources; Supervision; Writing‐review & editing.

## CONFLICT OF INTEREST

The authors declare no conflict of interest.

## Supporting information

Supplemental Table S1 Recent list of released and tested varieties tolerant to drought stress.
