# Supplementary material for: Developing drought‐smart, ready‐to‐grow future crops
Source: Plant Genome. 2022 Nov 10;16(1):e20279. doi: 10.1002/tpg2.20279 (PMC12807413; doi:10.1002/tpg2.20279)
Supplement: Supplementary file 1 [file TPG2-16-e20279-s001.docx]

**Supplementary Table 1** Recent list of released/tested varieties tolerant to drought stress.

| Plant | Variety name | Developed by | Reference |
| --- | --- | --- | --- |
| Chickpea (*Cicer arietinum* L.) | IPCMB 19-3, IPC L4-14 and BGM 4005 | International Crops Research Institute for the Semi-Arid Tropics (ICRISAT), India | ICRISAT, India |
| Jujube (*Ziziphus* L.) | Lingwuchangzao | Ningxia University, China | Jiang et al. (2020) |
| Alfalfa (*Medicago sativa* L.) | Longzhong | Gansu Agricultural University, China | Zhang et al. (2019) |
| Barley (*Hordeum vulgare* L.) | AC Metcalfe, CDC Copeland | United States Department of Agriculture, USA | Mahalingam (2017) |
| Barley (*Hordeum vulgare* L.) | Bentley | International Center for Genetic Engineering and Biotechnology (ICGEB), India | Tuteja et al. (2020) |
| Rice (*Oryza sativa* L.) | Swarnaprabha and Kattamodan | Kerala Agricultural University, India | Swapna and Shylaraj (2017) |
| Rice (*Oryza sativa* L.) | BRRI dhan 56 | ICGEB, India | Tuteja et al. (2020) |
| Wheat (*Triticum aestivum* L.) | Katya | Bulgarian Academy of Sciences, Bulgaria | Doneva et al. (2020) |
| Wheat (*Triticum aestivum* L.) | Atil | CIMMYT, Mexico | Guzmán et al. (2016) |
| Wheat (*Triticum aestivum* L.) | Willow creek | ICGEB, India | Tuteja et al. (2020) |
| Wheat (*Triticum aestivum* L.) | Ripper | ICGEB, India | Tuteja et al. (2020) |
| Maize (*Zea mays* L.) | Karoon | Shahid Beheshti University, Iran | Ahangir et al. (2020) |
| Sorghum (*Sorghum bicolor* L.) | P898012 | Selçuk University, Turkey | Akman et al. (2020) |
| Tomato (*Solanum lycopersicum* L.) | Varamin | Gorgan University of Agricultural Sciences and Natural Resources, Iran | Sousaraei et al. (2021) |
| Tomato (*Solanum lycopersicum* L.) | Zarina | Shandong Agricultural University, China | Zhang et al. (2020) |
| Finger millet (*Eleusine coracana* L.) | PR202 | G. B. Pant University of Agriculture and Technology, Pantnagar, India | Bartwal and Arora (2017) |
| Sugarcane (*Saccharum officinarum* L.) | Co 06022 | ICAR-Sugarcane Breeding Institute, Tamil Nadu, India | Devi et al. (2018) |

**References**

<https://www.cgiar.org/news-events/news/new-climate-resilient-disease-resistant-chickpea-varieties-coming-farmers-way/>

Ahangir, A., Ghotbi-Ravandi, A.A., Rezadoost, H., and Bernard, F. (2020). Drought tolerant maize cultivar accumulates putrescine in roots. *Rhizosphere***,** 100260.

Akman, H., Zhang, C., and Ejeta, G. (2020). Physio‐morphological, biochemical and anatomical traits of drought‐tolerant and susceptible sorghum cultivars under pre‐and post‐anthesis drought. *Physiologia Plantarum*.

Bartwal, A., and Arora, S. (2017). Drought stress-induced enzyme activity and mdar and apx gene expression in tolerant and susceptible genotypes of Eleusine coracana (L.). *In Vitro Cellular & Developmental Biology-Plant* 53(1)**,** 41-49.

Devi, K., Gomathi, R., Kumar, R.A., Manimekalai, R., and Selvi, A. (2018). Field tolerance and recovery potential of sugarcane varieties subjected to drought. *Indian Journal of Plant Physiology* 23(2)**,** 271-282.

Doneva, D., Pál, M., Brankova, L., Szalai, G., Tajti, J., Khalil, R., et al. (2020). The effects of putrescine pre‐treatment on osmotic stress responses in drought‐tolerant and drought‐sensitive wheat seedlings. *Physiologia plantarum*.

Guzmán, C., Autrique, J.E., Mondal, S., Singh, R.P., Govindan, V., Morales-Dorantes, A., et al. (2016). Response to drought and heat stress on wheat quality, with special emphasis on bread-making quality, in durum wheat. *Field Crops Research* 186**,** 157-165.

Jiang, W., Chen, L., Han, Y., Cao, B., and Song, L. (2020). Effects of elevated temperature and drought stress on fruit coloration in the jujube variety ‘Lingwuchangzao’(Ziziphus jujube cv. Lingwuchangzao). *Scientia Horticulturae* 274**,** 109667.

Mahalingam, R. (2017). Phenotypic, physiological and malt quality analyses of US barley varieties subjected to short periods of heat and drought stress. *Journal of cereal science* 76**,** 199-205.

Sousaraei, N., Mashayekhi, K., Mousavizadeh, S.J., Akbarpour, V., Medina, J., and Aliniaeifard, S. (2021). Screening of tomato landraces for drought tolerance based on growth and chlorophyll fluorescence analyses. *Horticulture, Environment, and Biotechnology***,** 1-15.

Swapna, S., and Shylaraj, K.S. (2017). Screening for osmotic stress responses in Rice varieties under drought condition. *Rice Science* 24(5)**,** 253-263.

Tuteja, N., Tuteja, R., Passricha, N., and Saifi, S. (2020). *Advancement in Crop Improvement Techniques.* Woodhead Publishing.

Zhang, C., Shi, S., Liu, Z., Yang, F., and Yin, G. (2019). Drought tolerance in alfalfa (Medicago sativa L.) varieties is associated with enhanced antioxidative protection and declined lipid peroxidation. *Journal of plant physiology* 232**,** 226-240.

Zhang, Z., Liu, Y., Cao, B., Chen, Z., and Xu, K. (2020). The effectiveness of grafting to improve drought tolerance in tomato. *Plant Growth Regulation* 91(1)**,** 157-167.
